# Tumor‐derived exosomal *BCYRN1* activates WNT5A/VEGF‐C/VEGFR3 feedforward loop to drive lymphatic metastasis of bladder cancer

**DOI:** 10.1002/ctm2.497

**Published:** 2021-07-19

**Authors:** Hanhao Zheng, Changhao Chen, Yuming Luo, Min Yu, Wang He, Mingjie An, Bowen Gao, Yao Kong, Yiyao Ya, Yan Lin, Yuting Li, Keji Xie, Jian Huang, Tianxin Lin

**Affiliations:** ^1^ Department of Urology Sun Yat‐sen Memorial Hospital Guangzhou Guangdong P. R. China; ^2^ Guangdong Provincial Key Laboratory of Malignant Tumor Epigenetics and Gene Regulation Sun Yat‐sen Memorial Hospital State Key Laboratory of Oncology in South China Guangzhou Guangdong P. R. China; ^3^ Department of General Surgery Guangdong Provincial People's Hospital Guangdong Academy of Medical Sciences Guangzhou Guangdong P. R. China; ^4^ Department of Pancreatobiliary Surgery Sun Yat‐sen Memorial Hospital Guangzhou Guangdong P. R. China; ^5^ Department of Urology Guangzhou First People's Hospital School of Medicine South China University of Technology Guangzhou China

**Keywords:** *BCYRN1*, bladder cancer, exosomes, lymph node metastasis, VEGF‐C/VEGFR3 signaling

## Abstract

**Background:**

Patients with lymph node (LN) metastatic bladder cancer (BCa) present with extremely poor prognosis. BCa‐derived exosomes function as crucial bioactive cargo carriers to mediate the signal transduction in tumor microenvironment triggering tumor metastasis. However, the mechanisms underlying exosome‐mediated LN metastasis in BCa are unclear.

**Methods:**

We conducted the high‐throughput sequencing to explore the expression profile of long noncoding RNA (lncRNA) in urinary exosomes (urinary‐EXO) from patients with BCa and further evaluated the clinical relevance of exosomal lncRNA *BCYRN1* in a larger 210‐case cohort. The functional role of exosomal *BCYRN1* was evaluated through the migration and tube formation assays *in vitro* and the footpad‐popliteal LN metastasis model *in vivo*. RNA pull‐down assays, luciferase assays, and actinomycin assays were conducted to detect the regulatory mechanism of exosomal *BCYRN1*.

**Results:**

LncRNA *BCYRN1* was substantially upregulated in urinary‐EXO from patients with BCa, and associated with the LN metastasis of BCa. We demonstrated that exosomal *BCYRN1* markedly promoted tube formation and migration of human lymphatic endothelial cells (HLECs) *in vitro* and lymphangiogenesis and LN metastasis of BCa *in vivo*. Mechanistically, *BCYRN1* epigenetically upregulated WNT5A expression by inducing hnRNPA1‐associated H3K4 trimethylation in WNT5A promoter, which activated Wnt/β‐catenin signaling to facilitate the secretion of VEGF‐C in BCa. Moreover, exosomal *BCYRN1* was transmitted to HLECs to stabilize the VEGFR3 mRNA and thus formed an hnRNPA1/WNT5A/VEGFR3 feedforward regulatory loop, ultimately promoting the lymphatic metastasis of BCa. Importantly, blocking VEGFR3 with specific inhibitor, SAR131675 significantly impaired exosomal *BCYRN1*‐induced the LN metastasis *in vivo*. Clinically, exosomal *BCYRN1* was positively associated with the shorter survival of BCa patients and identified as a poor prognostic factor of patients.

**Conclusion:**

Our results uncover a novel mechanism by which exosomal *BCYRN1* synergistically enhances VEGF‐C/VEGFR3 signaling‐induced lymphatic metastasis of BCa, indicating that *BCYRN1* may serve as an encouraging therapeutic target for patients with BCa.

## Graphical abstract

lncRNA *BCYRN1* is upregulated in urinary exosomes from bladder cancer (BCa) patients and associated with LN metastasis and poor prognosis of patients.

*BCYRN1* upregulates WNT5A expression to activate WNT/β‐catenin pathway and promotes the secretion of VEGF‐C in BCa.

Exosomal *BCYRN1* stabilizes VEGFR3 mRNA in HLECs, constituting a feedforward loop to promote lymphangiogenesis and LN metastasis of BCa.

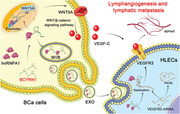

**Abbreviations**
3′UTR: 3′‐untranslated regions
ALIX: ALG‐2‐interacting protein X
BCa: bladder cancer
BLAST: basic local alignment search tool
CCK‐8: cell counting kit 8
ChIP: chromatin immunoprecipitation
ChIRP: chromatin isolation by RNA purification
CM: culture medium
DFS: disease‐free survival
ECM: endothelial cell medium
EdU: 5‐ethynyl‐20‐deoxyuridine
ELISA: enzyme‐linked immunosorbent assay
FBS: fetal bovine serum
FISH: fluorescence *in situ* hybridization
FRET: fluorescence resonance energy transfer
GSK3β: glycogen synthase kinase 3 beta
H3K4me3: H3K4 trimethylation
HDLECs: human dermal lymphatic endothelial cells
HLECs: human lymphatic endothelial cells
hnRNPA1: heterogeneous nuclear ribonucleoprotein A1
HUVECs: human umbilical vein endothelial cells
IHC: immunohistochemistry
ISH: *in situ* hybridization
IVIS: *in vivo* imaging system
LN: lymph node
lncRNA: long noncoding RNA
LYVE‐1: lymphatic vessel endothelial hyaluronan receptor 1
MS: mass spectrometry
NATs: normal adjacent tissues
NTA: nanoparticle tracking analysis
OS: overall survival
PBS: phosphate‐buffered saline
qRT‐PCR: quantitative real‐time reverse transcription polymerase chain reaction
RIP: RNA immunoprecipitation
RPMI: Roswell Park Memorial Institute
TCGA: The Cancer Genome Atlas
TEM: transmission electron microscopy
TFO: triplex‐forming oligonucleotides
TMEs: tumor microenvironments
TSG101: tumor susceptibility 101
TTS: triplex target sites
urinary‐EXO: urinary‐exosomes
VEGF‐C: vascular endothelial growth factor‐C
VEGFR3: vascular endothelial growth factor receptor 3
*χ* ^2^ tests: chi‐square tests

## 1 BACKGROUND

Bladder cancer (BCa) is one of the most frequently diagnosed urinary malignancies and is the second main reason of urinary cancer‐associated death worldwide.^1^ Tumor metastasis is the leading cause for the poor prognosis of patients with BCa, among which lymph node (LN) metastasis is recognized as the predominant metastatic manner.^2^, ^3^ Previous studies have demonstrated that patients with LN‐positive BCa have a worse 5‐year survival rate compared with patients with LN‐negative BCa, which decreased from 77.6% to 18.6%.^4^, ^5^ Despite the increasing evidences indicate that LN metastasis is a poor prognostic factor of BCa, the definite mechanisms driving LN metastasis of BCa are still unclear. Therefore, fully determining the molecular mechanisms triggering LN metastasis of BCa is of great clinical importance to explore efficient drug targets for therapeutic interventions in BCa patients.

The vascular endothelial growth factor‐C (VEGF‐C)/VEGF receptor 3 (VEGFR3) pathway is one of the most essential signaling for LN metastasis of tumors.^6^, ^7^ VEGF‐C induces dimerization and autophosphorylation of VEGFR3 to drive the differentiation of cardinal vein cells into lymphatic endothelial cells, resulting in the lymphangiogenesis of tumors.^8^, ^9^ It is well‐established that the VEGF‐C/VEGFR3 signaling is mediated by multiple biological processes during LN metastasis of tumors, including transcriptional regulation, chemokine induction, and the crosstalk between signaling pathways.^10^, ^11^, ^12^ Transducin (β)‐like 1 X‐linked receptor 1 transcriptionally promotes VEGF‐C expression through binding with its promoter, thus enhancing lymphatic metastasis of esophageal carcinoma.^13^ Inhibition of tumor necrosis factor alpha‐activated inflammatory macrophage‐secreted VEGF‐C is identified as a promising intervention for lymphangiogenesis of tumors.^14^ Nevertheless, the mechanisms underlying the coordinate stimulation of VEGF‐C/VEGFR3 signaling leading to LN metastasis of BCa are largely unknown.

Exosomes are nano‐sized lipid bilayer microvesicles ranging from 30 to 150 nm in diameter that possess tissue‐specific anchoring proteins in the transmembrane, enabling their highly targeted endocytosis by recipient cells.^15^ Tumor‐derived exosomes served as crucial cargo carriers of bioactive molecules contribute to the trafficking between tumor cells and the tumor microenvironment (TME).^16^, ^17^ Transmission of exosomes from tumor cells to establish the premetastatic niche is a vital step in the metastatic cascade resulting in cancer dissemination.^18^, ^19^ Exosomes transfer the miR‐221‐3p to lymphatic endothelial cells to remodel the TME, inducing the LN metastasis of cervical squamous cell carcinoma.^20^ Moreover, targeting exosomal contents to inhibit macrophage polarization is an attractive method to prevent tumor metastasis.^21^ However, the regulatory mechanisms of exosomes in lymphatic metastasis of BCa remain confusing.

In the present study, we conducted a high‐throughput sequencing to identify a long noncoding RNA (lncRNA) *BCYRN1*, which was significantly overexpressed in urinary exosomes (urinary‐EXO) from patients with BCa and was closely associated with LN metastasis and poor prognosis of patients. We demonstrated that exosomal *BCYRN1* enhanced lymphangiogenesis and LN metastasis of BCa *in vitro* and *in vivo*. Mechanistically, exosomal *BCYRN1* constituted a feedforward loop with hnRNPA1/WNT5A/VEGFR3 regulatory axis and synergistically enforced the VEGF‐C/VEGFR3 signaling to facilitate the lymphangiogenesis of BCa. Our results uncover the precise mechanism by which exosomal *BCYRN1* promotes lymphatic metastasis of BCa, implying that targeting *BCYRN1* may become an encouraging therapy for patients with LN metastatic BCa.

## 2 MATERIALS AND METHODS

### 2.1 Clinical specimens

The tissues and urine specimens were obtained from patients in Sun Yat‐sen Memorial Hospital, Sun Yat‐sen University (Guangzhou, China). Pathologically, tissues specimens were verified as BCa through two independent professional pathologists. The clinical characteristics of the participants were listed in Table S1. We obtained the informed consent from involved patients and the ethical approvement of the Committees for Ethical Review of Research Involving Human Subjects at Sun Yat‐sen University (approval number:2013[61]).

### 2.2 Cell culture

All cell lines involved in our study except human lymphatic endothelial cells (HLECs), human dermal lymphatic endothelial cells (HDLECs), and human umbilical vein endothelial cells (HUVECs) were purchased from American Type Culture Collection (ATCC, Manassas, VA, USA). T24 and 5637 cells were cultured in Roswell Park Memorial Institute 1640 (Gibco, Shanghai, China), UM‐UC‐3 cells in Dulbecco's modified Eagle's medium (Gibco, Shanghai, China), and SVHUC‐1 cells in F‐12K medium (Hyclone, Logan, UT, USA). All media were supplemented with 10% fetal bovine serum (FBS) (Gibco, Shanghai, China). HLECs were purchased from ScienCell Research Laboratories (Carlsbad, CA, USA) while HDLECs and HUVECs from Promocell company (Heidelberg, Germany), both of which were kept in endothelial cell medium with 5% FBS and 1% matched growth factor (ScienCell, CA, USA). All cell lines mentioned above were cultured in a humidified incubator at 37°C with 5% CO_2_.

### 2.3 RNA sequencing and data analysis

The extraction of total RNA in the urinary‐EXO isolated from five patients with muscle invasive BCa and five healthy controls was conducted with TRIZOL (Invitrogen, Carlsbad, CA, USA). The RNA was qualified by Agilent 2100 bioanalyzer (Thermo Fisher Scientific, Waltham, MA, USA), after which the qualified RNA was subjected to remove rRNA through a Ribo‐Zero Magnetic Kit (Epicentre) and sequenced on a Hiseq4000 platform.

### 2.4 Mouse popliteal LN metastasis model

The BALB/c nude mice (about 4‐ to 5‐week‐old) were purchased form the Experimental Animal Center, Sun Yat‐sen University (Guangzhou, China) and used to construct the footpad tumor model as described in our previous study,^10^ in which 5 × 10^5^ UM‐UC‐3 cells supplemented in 20 μl phosphate buffered saline (PBS) and 10 μg indicated exosomes supplemented in 20 μl PBS were slowly injected into the footpad of mice, respectively. Then, the footpad tumors and popliteal LNs were resected and embedded with paraffin for further analysis. The experiments were carried out under the approval of the Institutional Animal Care and Use Committee of Sun Yat‐sen University.

### 2.5 RNA pull‐down assays

To examine *BCYRN1*‐interating proteins, biotin RNA pull‐down assays were conducted. Transcript Aid T7 High Yield Transcription Kit and Pierce Magnetic RNA‐Protein Pull‐Down Kit (Thermo Fisher Scientific) were used to synthesize biotinylated *BCYRN1* and captured the *BCYRN1* binding proteins, respectively.

### 2.6 Chromatin isolation by RNA purification (ChIRP) assays

The ChIRP assays were carried out through a Magna ChIRP RNA Interactome Kit (Millipore, Billerica, MA, USA) with instructions of the manufacturers. The *BCYRN1*‐specific probes were designed with an online tool and labeled with biotin in its 3′ terminus (Table S9).

### 2.7 Fluorescence resonance energy transfer analysis

To verify the triplex structure forming between *BCYRN1* and *WNT5A* promoter, the fluorescence resonance energy transfer (FRET) analysis was conducted, in which the triplex‐forming oligonucleotides (TFO) in *BCYRN1* were labeled with 5‐carboxytetramethylrhodamine, and the triplex target site (TTS) in *WNT5A* promoter was labeled with 5‐carboxyfluorescein. Then, the binding buffer added with TFO and TTS was incubated at 55°C for 10 min and 37°C for 10 h, after which the fluorescence wavelengths were detected with a Molecular Device M5 Plate Reader.

### 2.8 Chromatin immunoprecipitation (ChIP) assays

The chromatin immunoprecipitation (ChIP) assays were conducted to detect the interaction between hnRNPA1 or H3K4me3 and their target chromatins with EZ‐Magna ChIP A/G kit (Millipore). Briefly, BCa cells were harvested to fixe in 4% paraformaldehyde for 10 min after which the fixed cells were resuspended in cell lysis buffer to thoroughly lyse the cells. Then, the chromatins of cell lysate were sheared by sonicator and added into the mixtures of primary antibodies and magnetic beads for further incubation at 4°C overnight. Finally, the binding chromatins were eluted and further analyzed through quantitative real‐time reverse transcription polymerase chain reaction (qRT‐PCR) assays.

### 2.9 CRISPR/Cas9‐mediated gene deletion

The lentiCRISPR v2 vectors contain with single guide RNAs targeted the *BCYRN1* were purchased from GenePharma (Shanghai, China) and further transfected into HLECs to construct the *BCYRN1*‐knockouting HLECs. qRT‐PCR analysis was performed to evaluate the knockout efficiency.

### 2.10 Further applied methods

Further methods of immunohistochemistry (IHC) analysis, *in situ* hybridization (ISH) analysis, cytosolic and nuclear fraction, immunofluorescence, fluorescence *in situ* hybridization (FISH), isolation of exosomes, transmission electron microscopy (TEM) lentivirus infection and cell transfection, 5‐ethynyl‐20‐deoxyuridine (EdU) assays, Cell Counting Kit 8 (CCK‐8) assays, subcutaneous tumorigenicity model, tube formation and Transwell assays, western blotting analysis, RNA extraction, RNA immunoprecipitation (RIP) assays, serial deletion analysis, TOP‐flash/FOP‐flash assays, dual‐luciferase reporter assays, enzyme‐linked immunosorbent assay (ELISA), internalization analysis of exosomes, actinomycin assays and bioinformatic analysis were provided in Supplementary Methods.

### 2.11 Statistical analysis

Statistical analysis was carried out using SPSS v.13.0 (SPSS Inc., Chicago, IL, USA), and *p* < 0.05 was considered as statistically significant. All quantitative data performed in triplicate were expressed as mean ± SD. Kaplan–Meier analysis was carried out to evaluate the survival time of patients with BCa. Moreover, chi‐square tests (*χ* ^2^ tests) and one‐way ANOVA or two‐tailed Student's *t*‐test were performed to assess the statistical significance between non‐parametric variables and parametric variables, respectively.

## 3 RESULTS

### 3.1 Exosomal *BCYRN1* is correlated with LN metastasis of BCa

**FIGURE 1:**
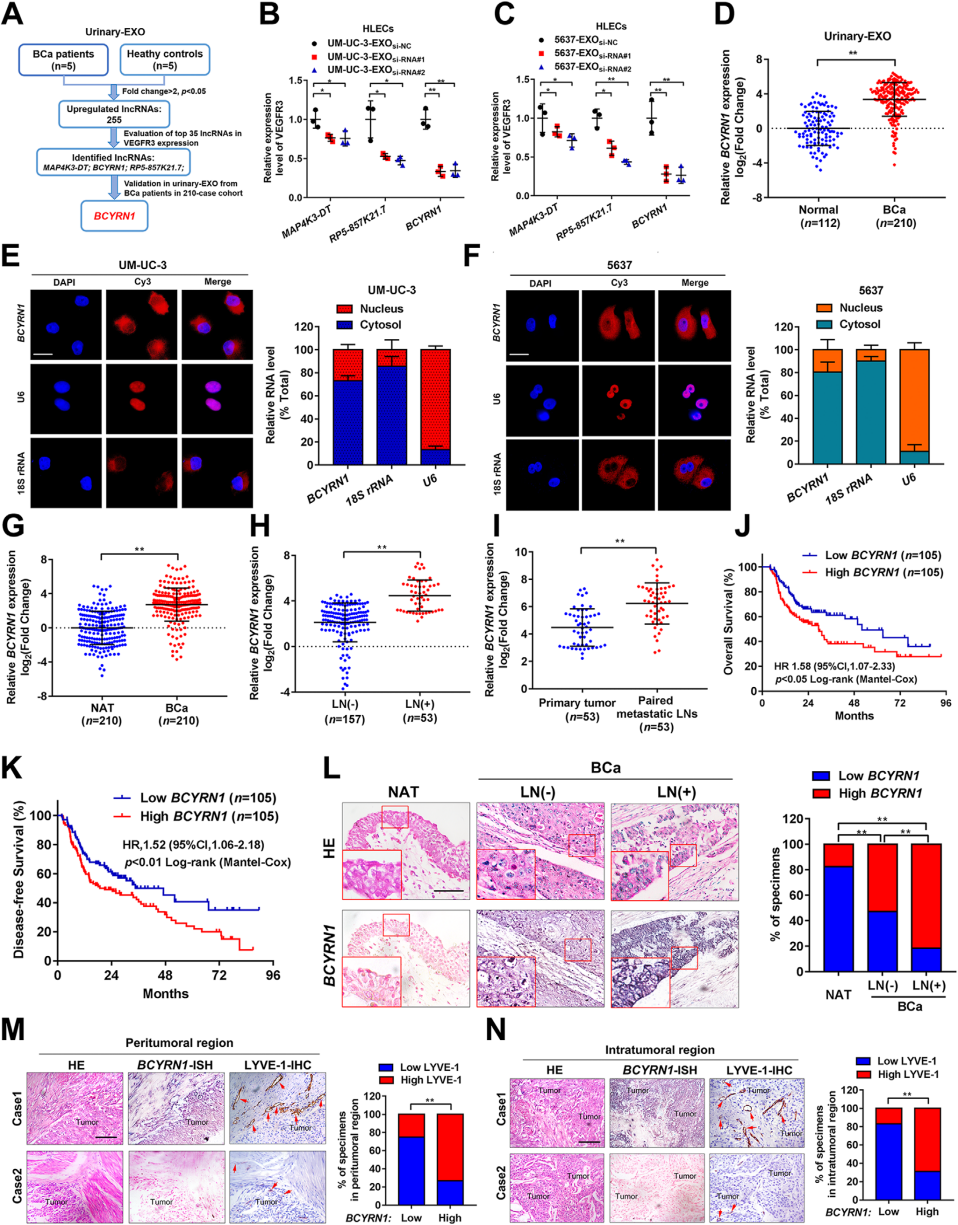
Exosomal *BCYRN1* overexpression correlates with LN metastasis of BCa. (A) Flow chart for the identification of exosomal *BCYRN1* overexpression in urinary‐EXO from patients with BCa and correlated with VEGFR3 expression. (B and C) qRT‐PCR analysis of VEGFR3 expression in HLECs treated with relative lncRNA‐silenced BCa cells‐secreted exosomes. (D) qRT‐PCR analysis verified the overexpression of *BCYRN1* in urinary‐EXO from 210 patients with BCa compared with those from 112 healthy controls. (E and F) FISH and nuclear fractionation analysis of *BCYRN1* subcellular allocation in UM‐UC‐3 and 5637 cells. Scale bars: 5 μm. (G) qRT‐PCR analysis of *BCYRN1* expression in BCa tissues and NATs in a 210‐case cohort. (H and I) Comparison of *BCYRN1* expression in LN‐positive and LN‐negative BCa tissues and primary tumor tissues and paired metastatic LNs. (J and K) Kaplan‐Meier survival analysis of the OS and DFS for patients with BCa with low versus high *BCYRN1* expression. The median *BCYRN1* expression was used as the cutoff value. (L) Representative images and percentages of *BCYRN1* expression in NATs, and LN‐negative and LN‐positive BCa tissues from ISH analysis. Scale bars: 50 μm. (M and N) Representative images and percentages of *BCYRN1* expression and LYVE‐1‐indicated lymphatic vessels in peritumoral and intratumoral regions of BCa tissues from ISH and IHC analyses. Scale bars: 50 μm. The statistical difference was assessed through one‐way ANOVA followed by Dunnett's tests in B and C; the nonparametric Mann–Whitney *U* test in D, G‐I; and the χ2 test in L‐N. Error bars show the standard deviations derived from three independent experiments. *p < 0.05; **p < 0.01

It has been proposed that targeting exosome‐dependent transmission of specific lncRNA to a prearranged cell represents a promising clinical intervention to inhibit tumor metastasis.^22^ However, its role in LN metastasis of BCa is still largely unknown. To determine the mechanism and clinical application potential of exosomal lncRNA in LN metastasis of BCa, we first conducted high‐throughput sequencing to explore the lncRNA expression profile of urinary‐EXO (GEO ID: GSE156308). As shown in Figure 1A, 255 lncRNAs were overexpressed in the urinary‐EXO from BCa patients compared with those from healthy controls. Next, we evaluated the essential exosomal lncRNA that correlated with the activation of the VEGF‐C/VEGFR3 signaling which is the most prominent manner in stimulating LN metastasis of tumors.^6^ The results revealed that three lncRNA, *BCYRN1, MAP4K3‐DT*, and *RP5‐857K21.7* were positively associated with the VEGF‐C/VEGFR3‐induced LN metastasis of BCa (Figures 1B and 1C and Table S2). Further validation in our larger 210‐case clinical cohort confirmed that *BCYRN1* was the most significantly overexpressed lncRNA in urinary‐EXO from patients with BCa as compared with those from healthy controls (Figure 1D).

Using FISH and subcellular fractionation assays, we found that *BCYRN1* was detected in both cytoplasm and nucleus of UM‐UC‐3 and 5637 cells (Figures 1E and 1F). Moreover, statistical analysis of The Cancer Genome Atlas database demonstrated that *BCYRN1* was strikingly overexpressed in multiple cancers and is associated with poor prognosis of patients (Figures S1A‐S1I).

To further explore the clinical role of *BCYRN1* in BCa, qRT‐PCR analysis was conducted and revealed that *BCYRN1* was markedly overexpressed in BCa tissues as compared with paired normal adjacent tissues (NATs) (Figure 1G). As shown in Figure 1H and Table S1, *BCYRN1* overexpression in BCa was related to the LN metastasis of patients. Furthermore, *BCYRN1* expression in metastatic LN was markedly higher than that in corresponding primary tumors (Figure 1I), indicating that *BCYRN1* is a vital constituent in LN metastatic BCa cells. Kaplan–Meier analysis showed that *BCYRN1* overexpression was positively related to the poor prognosis of BCa patients (Figures 1J and 1K). Strikingly, ISH analysis showed that *BCYRN1* was hardly detected in NATs and slightly increased in BCa tissues without LN metastasis, while was significantly upregulated in BCa tissues with LN metastasis (Figure 1L). A positive correlation between the expression of *BCYRN1* and density of microlymphatic vessel was observed in both peritumoral and intratumoral regions of BCa tissues, as indicated by a positive signal of lymphatic vessel endothelial hyaluronan receptor 1 (LYVE‐1) in IHC analysis (Figures 1M and 1N), suggesting that *BCYRN1* may involve in the lymphangiogenesis of BCa. Taken together, these findings indicate that *BCYRN1* contributes to the LN metastasis and poor prognosis of patients with BCa.

### 3.2 *BCYRN1* is overexpressed in BCa cell‐secreted exosomes

**FIGURE 2:**
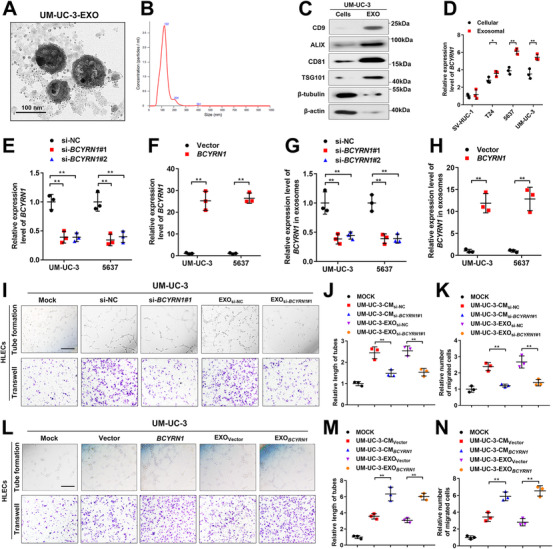
Exosomal *BCYRN1* promotes the lymphangiogenesis of BCa *in vitro*. (A and B) The characteristics of exosomes isolated from UM‐UC‐3 cells culture media were identified by TEM and NTA. Scale bars: 100 nm. (C) Western blotting analysis of the indicated exosomal markers in the UM‐UC‐3 cell lysate and purified exosomes from the culture media. (D) qRT‐PCR analysis of *BCYRN1* expression in SVHUC‐1 and BCa cell lines (T24, UM‐UC‐3, 5637) and their corresponding exosomes isolated from the culture media. (E and F) qRT‐PCR analysis of *BCYRN1* expression in UM‐UC‐3 and 5637 cells after silencing or overexpressing *BCYRN1*. (G and H) qRT‐PCR analysis of *BCYRN1* levels in exosomes isolated from *BCYRN1*‐silenced or overexpressing UM‐UC‐3 and 5637 cells culture media. (I‐K) Representative images and quantification of tube formation and Transwell migration for HLECs treated with PBS, culture media from UM‐UC‐3_si‐NC_ or UM‐UC‐3_si‐_ *_BCYRN1_*_#1_ cells, and their corresponding exosomes. Scale bars: 100 μm. (L‐N) Representative images and quantification of tube formation and Transwell migration for HLECs treated with PBS, culture media from UM‐UC‐3_Vector_ or UM‐UC‐3*_BCYRN1_* cells, and their corresponding exosomes. Scale bars: 100 μm. The statistical difference was assessed through one‐way ANOVA followed by Dunnett's tests in D, E, G, J, K, M, and N; and two‐tailed Student's *t* test in F and H. Error bars show the standard deviations derived from three independent experiments. *p < 0.05; **p < 0.01

Since we observed that *BCYRN1* was remarkably overexpressed in the urinary‐EXO from patients with BCa, the level of *BCYRN1* in exosomes secreted by BCa cells was further determined. First, we collected the culture medium (CM) of BCa cells to isolate and purify the exosomes. TEM and nanoparticle tracking analysis (NTA) were performed to show the cup‐shaped structure of isolated particles with a size distribution of 30–150 nm (Figures 2A, 2B, and S1J). Western blotting analysis detected the enrichment of typical exosomal protein markers CD9, ALG‐2‐interacting protein X (ALIX), CD81 and Tumor Susceptibility 101 (TSG101) in the isolated particles but not in the cell extracts (Figures 2C and S1K), confirming that the isolated particles are exosomes. Subsequently, qRT‐PCR analysis revealed that *BCYRN1* levels were significantly increased in BCa cell‐secreted exosomes compared with that in exosomes secreted by human normal bladder epithelial cells (SVHUC‐1) (Figure 2D). Intriguingly, a higher level of *BCYRN1* was found in BCa cell‐secreted exosomes compared with its intracellular level (Figure 2D), suggesting that *BCYRN1* may function preferentially via exosomal transmission. Overexpressing *BCYRN1* in BCa cells significantly increased the *BCYRN1* level in relative exosomes, while silencing *BCYRN1* decreased its levels in corresponding exosomes (Figures 2E–2H), indicating that altering the intracellular *BCYRN1* expression has an obvious effect in the level of exosomal *BCYRN1*. Collectively, our results reveal that *BCYRN1* is overexpressed in exosomes secreted by BCa cells.

### 3.3 Exosomal *BCYRN1* enhances lymphangiogenesis of BCa *in vitro*

Lymphangiogenesis is well‐characterized as the rate‐limiting process of tumor LN metastasis.^23^ Our results showed that *BCYRN1* overexpression positively correlated with lymphangiogenesis in BCa tissues. Therefore, the tube formation and migration assays were performed to explore the regulatory function of *BCYRN1* in lymphangiogenesis of BCa. As shown in Figures 2I–2K and S2A, the tube formation and migration of HLECs were significantly inhibited after incubating with the CM from *BCYRN1*‐silenced UM‐UC‐3 and 5637 cells. Importantly, we found that silencing *BCYRN1* attenuated the ability of exosomes secreted by UM‐UC‐3 and 5637 cells to enhance the tube formation and migration of HLECs, the results of which were confirmed by another lymphatic endothelial cell lines, HDLECs (Figures 2I–2K and S2A and S2B). Conversely, the CM and isolated exosomes from *BCYRN1*‐overexpressing BCa cells dramatically increased the tube formation and migration of both HLECs and HDLECs (Figures 2L–2N and S2C and S2D). Since tumor angiogenesis is also crucial for tumor metastasis,^24^ we further evaluated the role of exosomal *BCYRN1* in the angiogenesis of BCa by conducting the tube formation and migration assays using HUVECs, which revealed that the CM and isolated exosomes from *BCYRN1*‐silenced or overexpressing UM‐UC‐3 cells have no significant effect on the tube formation and migration of HUVECs (Figures S3A‐S3F). Moreover, silencing *BCYRN1* markedly inhibited the proliferation of BCa cells as determined by EdU and CCK‐8 assays, while no obvious effect of silencing *BCYRN1* on the invasion of BCa cells was observed (Figures S4A‐S4F), further confirming that exosomal *BCYRN1* contributes to the LN metastasis of BCa through mediating the lymphangiogenesis. Collectively, these findings reveal that overexpression of exosomal *BCYRN1* promotes the lymphangiogenesis of BCa *in vitro*.

### 3.4 Exosomal *BCYRN1* promotes the LN metastasis of BCa *in vivo*

**FIGURE 3:**
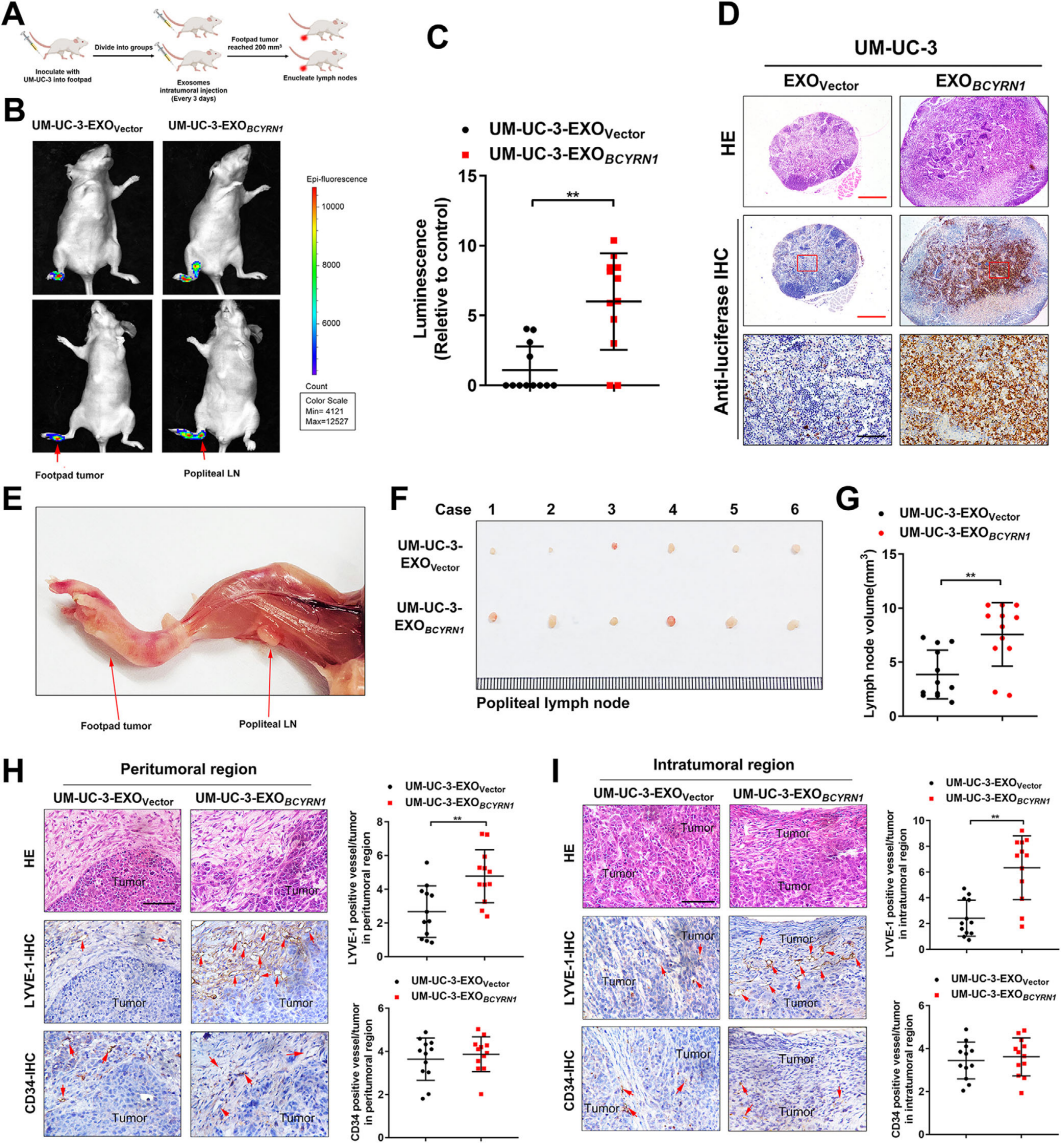
Exosomal *BCYRN1* enhances LN metastasis of BCa *in vivo*. (A) Schematic diagram for the construction of the nude mice popliteal LN metastasis model. (B and C) Representative images and quantification of bioluminescence for popliteal LN in the nude mice model intratumorally injected with UM‐UC‐3‐EXO_Vector_ or UM‐UC‐3‐EXO*_BCYRN1_* (*n* = 12). (D) Representative images of anti‐luciferase IHC analysis for popliteal LN in UM‐UC‐3‐EXO_Vector_ or UM‐UC‐3‐EXO*_BCYRN1_*‐treated nude mice (*n* = 12). Scale bars: 50 μm. (E) Representative image of a popliteal LN in the nude mice model. (F) Representative image of the enucleated popliteal LNs in UM‐UC‐3‐EXO_Vector_ or UM‐UC‐3‐EXO*_BCYRN1_*‐treated nude mice (*n* = 12). (G) The volume for the popliteal LN in nude mice model treated with UM‐UC‐3‐EXO_Vector_ or UM‐UC‐3‐EXO*_BCYRN1_* (*n* = 12). (H and I) Representative images and percentages of LYVE‐1‐indicated lymphatic vessel or CD34‐indicated blood vessel density in peritumoral and intratumoral regions of footpad primary tumor tissues from the IHC analysis. Scale bars: 50 μm. The statistical difference was assessed through two‐tailed Student's *t* test in C, G, H, and I. Error bars show the standard deviations derived from three independent experiments. *p < 0.05; **p < 0.01

Next, we examined the impact of exosomal *BCYRN1* in the LN metastasis of BCa *in vivo*. Since tumorigenicity is vital for tumor LN metastasis,^6^ we first evaluated the oncogenic function of *BCYRN1* in the tumorigenicity of BCa by constructing the subcutaneous xenograft model. The results showed that the tumor volume in sh‐*BCYRN1*#1 group was significantly smaller than that in sh‐NC group (Figures S5A and S5B). Higher expression level of Ki‐67 was observed in the sh‐NC group compared with sh‐*BCYRN1*#1 group (Figures S5C and S5D), indicating that *BCYRN1* contributed to the tumorigenicity of BCa. To further detect the regulatory function of exosomal *BCYRN1* in LN metastasis, a footpad‐popliteal LN metastasis model was established. UM‐UC‐3 cells labeled with luciferase were implanted into the footpad of nude mice, after which the mice were randomly assigned to two groups following with the treatment of equivalent vector plasmid‐transfected UM‐UC‐3 cell‐secreted exosomes (UM‐UC‐3‐EXO_Vector_) or *BCYRN1* plasmid‐transfected UM‐UC‐3 cell‐secreted exosomes (UM‐UC‐3‐EXO*_BCYRN1_*) (Figure 3A). Strikingly, UM‐UC‐3‐EXO*_BCYRN1_* injection prominently enhanced the metastasis of the primary tumor to the popliteal LNs of nude mice compared with the group treated with UM‐UC‐3‐EXO_Vector_, as detected using an *In Vivo* Imaging System (IVIS) (Figures 3B and 3C and S5E‐S5G). We found that UM‐UC‐3‐EXO*_BCYRN1_* significantly enlarged the volume of popliteal LNs and increased the rate of LN metastasis (Figures 3D–3G, Tables S3 and S4). IHC analysis showed a higher density of lymphatic vessels in UM‐UC‐3‐EXO*_BCYRN1_*‐treated group than UM‐UC‐3‐EXO_Vector_‐treated group, whereas no significant difference of blood vessels density as indicated by CD34 was observed between these two groups (Figures 3H and 3I), suggesting that exosomal *BCYRN1* markedly induces lymphangiogenesis *in vivo*. Collectively, these findings reveal that exosomal *BCYRN1* enhances the lymphangiogenesis and LN metastasis of BCa *in vivo*.

### 3.5 *BCYRN1* directly binds with hnRNPA1

**FIGURE 4:**
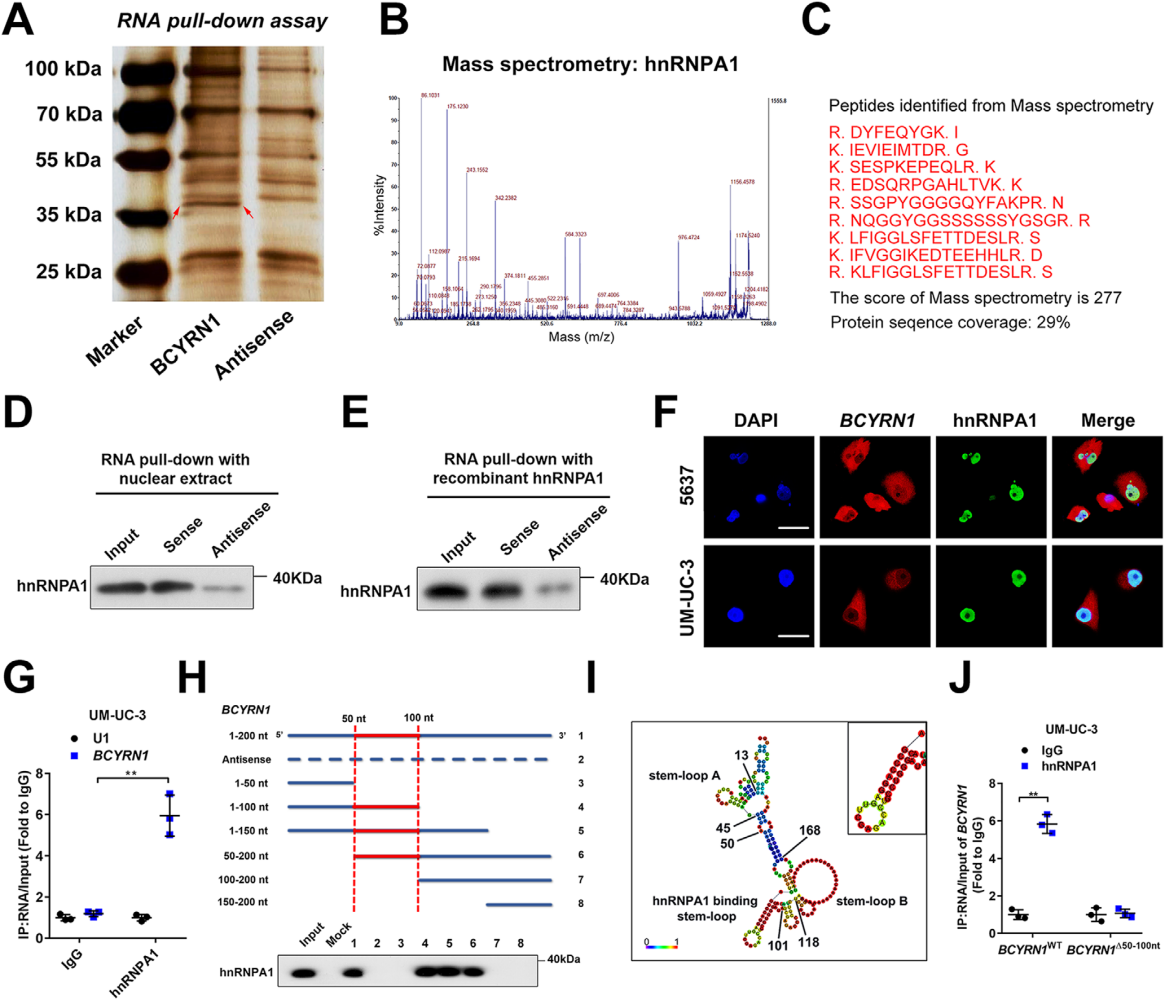
*BCYRN1* directly interacts with hnRNPA1. (A) The image of silver staining for RNA pull‐down assay with *BCYRN1* sense and antisense RNAs. (B and C) Mass spectrometry analysis of *BCYRN1*‐binding proteins after RNA pull‐down assay. (D and E) Western blotting analysis after RNA pull‐down with nuclear extract or recombinant hnRNPA1. (F) Representative images of immunofluorescence of *BCYRN1* and hnRNPA1 in 5637 and UM‐UC‐3 cells. Scale bars: 5 μm. (G) The enrichment of *BCYRN1* by the anti‐hnRNPA1 antibody after RIP assays in UM‐UC‐3 cells. IgG was used as negative control and U1 as non‐specific control. (H) Western blotting analysis of RNA pull‐down with serial deletions of *BCYRN1*. (I) The prediction showed the stem‐loop structure of hnRNPA1 binding sequences in *BCYRN1*. (J) qRT‐PCR analysis of RIP assays after deleting the 50–100‐nt regions of *BCYRN1* in UM‐UC‐3 cells. The statistical difference was assessed through a two‐tailed Student's *t* test in G and J. Error bars show the standard deviations derived from three independent experiments. *p < 0.05; **p < 0.01

Considering that lncRNAs frequently exert its biological functions in cellular processes by interacting with proteins,^25^ we conducted RNA pull‐down assays using biotinylated *BCYRN1* and antisense *BCYRN1* as control to determine the *BCYRN1* interacting proteins in BCa cells. The results showed that an apparent band in 35–40 kDa was enriched by biotinylated *BCYRN1*, which was validated as heterogeneous nuclear ribonucleoprotein A1 (hnRNPA1) using mass spectrometry (MS) (Figures 4A–4C). Western blotting analysis after RNA pull‐down assays confirmed the interaction between *BCYRN1* and hnRNPA1 (Figures 4D and 4E). Confocal microscopy revealed the co‐localization of *BCYRN1* and hnRNPA1 in 5637 and UM‐UC‐3 cells (Figure 4F). RIP assays were implemented to show that *BCYRN1* was obviously enriched by hnRNPA1 compared with the negative control (Figures 4G and S6A), further demonstrating that *BCYRN1* directly binds with hnRNPA1. Moreover, serial deletion assays confirmed that the 50‐100‐nt sequence in the 5′‐terminal of *BCYRN1* was essential for its interaction with hnRNPA1 (Figure 4H). We next utilized POSTAR2, a website for predicting the protein‐RNA interactions,^26^ to predict the hnRNPA1 potential binding sequence motif located on the 50‐100‐nt region in the 5′‐terminal of *BCYRN1*, which formed a stem‐loop structure (Figures 4I and S6B‐S6D). RIP assays showed that the enrichment of *BCYRN1* by hnRNPA1 was obviously attenuated after directly deleting the 50–100‐nt region in the 5′‐terminal of *BCYRN1* (Figures 4J and S6E). Collectively, these results demonstrate the direct interaction between hnRNPA1 and the 50‐100‐nt region in 5′‐terminal of *BCYRN1*.

### 3.6 *BCYRN1* upregulates WNT5A expression by forming a DNA‐RNA triplex with its promoter

**FIGURE 5:**
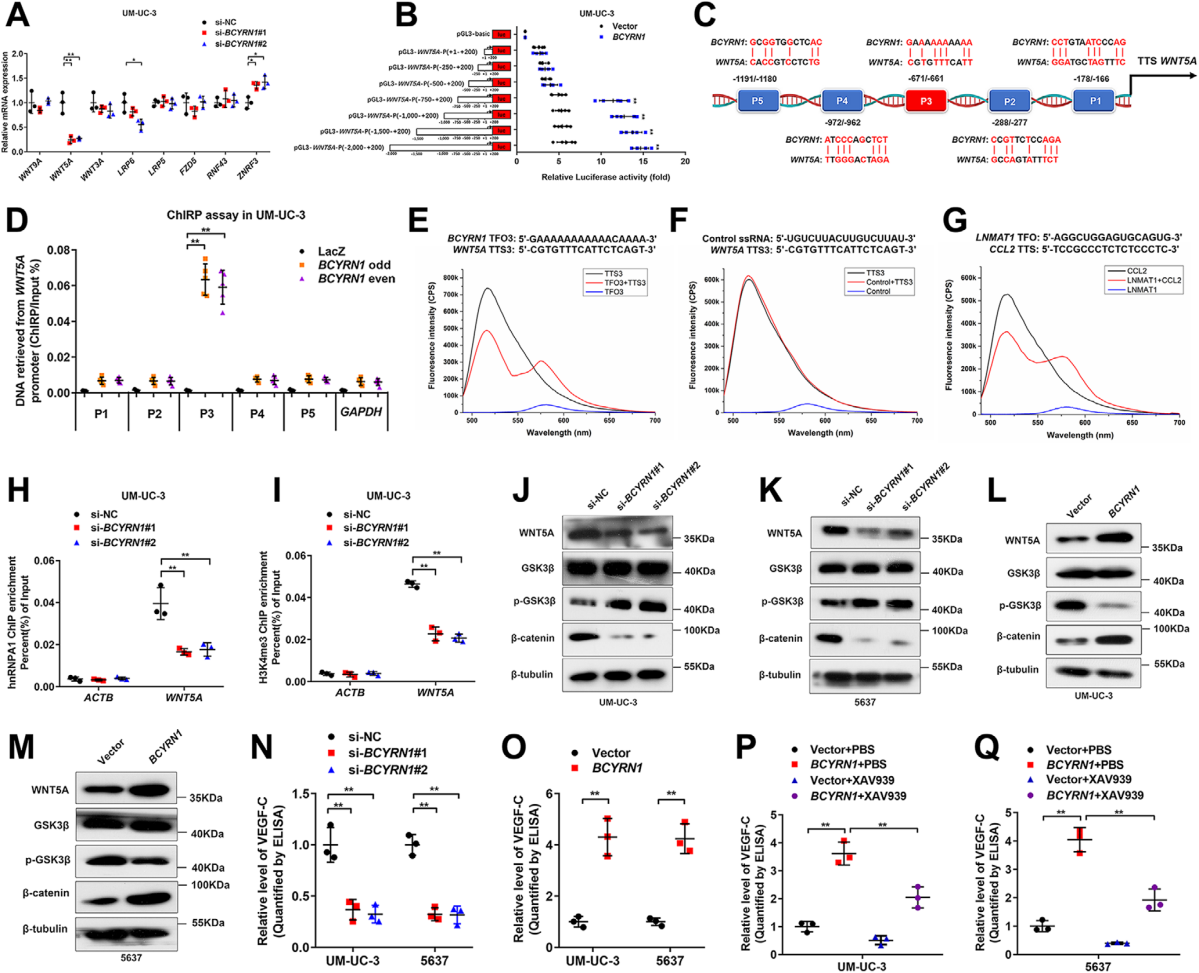
*BCYRN1* epigenetically upregulates *WNT5A* expression to activate the Wnt/β‐catenin pathway and promote the secretion of VEGF‐C in BCa cells. (A) Core genes involved in the Wnt/β‐catenin pathway were detected using qRT‐PCR analysis in *BCYRN1*‐silenced UM‐UC‐3 cells. (B) Luciferase activity was detected in *BCYRN1*‐overexpressing UM‐UC‐3 cells transfected with truncate *WNT5A* promoter plasmids. (C) Schematic diagram of the predicted *BCYRN1* binding sites in the *WNT5A* promoter. (D) ChIRP assays detected the *BCYRN1*‐associated chromatin in UM‐UC‐3 cells. (E‐G) FRET analysis of TFO in *BCYRN1* with TTS in *WNT5A* promoter. The Control ssRNA with TTS in *WNT5A* promoter was used as negative control and the TFO in *LNMAT1* with TTS in *CCL2* promoter as positive control. (H and I) ChIP‐qPCR analysis evaluated the hnRNPA1 occupancy and H3K4me3 status in the *WNT5A* promoter in *BCYRN1*‐silenced UM‐UC‐3 cells. (J‐M) Western blotting analysis evaluated the activation of the Wnt/β‐catenin pathway by *BCYRN1* overexpression in UM‐UC‐3 cells. (N and O) The secretion of VEGF‐C from UM‐UC‐3 and 5637 cells after silencing or overexpressing *BCYRN1*, as evaluated using ELISA. (P and Q) ELISA for XAV939 treatment on *BCYRN1* overexpression‐induced VEGF‐C secretion by UM‐UC‐3 and 5637 cells. The statistical difference was assessed through one‐way ANOVA followed by Dunnett's tests in A, B, D, H, I, N, P and Q; and two‐tailed Student's *t* test in O. Error bars show the standard deviations derived from three independent experiments. *p < 0.05; **p < 0.01

In light of the emerging evidence that lncRNAs are widely participated in the regulation of cellular signaling pathways in tumor progression,^27^, ^28^ we detected the alteration of essential signaling‐related gene after silencing or overexpressing the *BCYRN1* in BCa cells. The results showed that *WNT5A* was the most obviously altered gene that correlated with *BCYRN1* expression, in which it was dramatically downregulated in *BCYRN1*‐silenced BCa cells and upregulated in *BCYRN1*‐overexpressing BCa cells (Figures 5A and S6F‐S6H). Moreover, luciferase assays revealed that transfecting the plasmids containing −500 to −750 bp fragments of the *WNT5A* promoter significantly increased the luciferase activity in *BCYRN1*‐overexpressing BCa cells (Figures 5B and Figure S6I). ChIRP assays showed that *BCYRN1* directly bound to the region of −661 to −671 bp in *WNT5A* promoter (Figures 5C and 5D and S6J). In addition, we used a prediction tool for the binding motifs between lncRNA and DNA, LongTarget,^29^ to predict five potential TFOs in *BCYRN1* and the corresponding TTS in *WNT5A* promoter (Table S5). The FRET analysis was performed to show a remarkable increase of the fluorescence intensity at 570–580 nm and a reduction at 520 nm in the *BCYRN1* TFO3/*WNT5A* TTS3 group as compared with single‐stranded RNA/*WNT5A* TTS3 control group, which was consistent with that in the *LNMAT1* TFO/*CCL2* TTS positive control group^11^ (Figures 5E–5G). To further determine the essential role of −661 to −671 bp region of *WNT5A* promoter in *BCYRN1*‐induced transactivation of *WNT5A*, the luciferase assays were performed and revealed that the luciferase activity of *WNT5A* promoter was markedly reduced by silencing *BCYRN1* and increased by overexpressing *BCYRN1*, while mutating the *WNT5A* promoter (*WNT5A*‐P3) impaired the *BCYRN1* induction of luciferase activity of the *WNT5A* promoter (Figures S6K‐S6N). We previously demonstrated that hnRNPA1‐induced H3K4 trimethylation (H3K4me3) plays a vital role in the epigenetic reprogramming contributing to the regulation of gene expression.^30^, ^31^ Therefore, we evaluated whether *BCYRN1* mediated the transcriptional activation of *WNT5A* by increasing the hnRNPA1‐induced H3K4me3 on *WNT5A* promoter. ChIP‐qPCR analysis showed that the enrichment of hnRNPA1 and H3K4me3 status in *WNT5A* promoter were significantly reduced after silencing *BCYRN1*, while overexpressing *BCYRN1* dramatically increased the occupancy of hnRNPA1 and H3K4me3 in *WNT5A* promoter in BCa (Figures 5H and 5I and S7A‐S7F). Moreover, silencing hnRNPA1 significantly impaired *BCYRN1*‐induced H3K4me3 enrichment in *WNT5A* promoter and transcriptional activation of *WNT5A* in BCa cells (Figures S7G‐S7J). Taken together, our findings verify that *BCYRN1* activates *WNT5A* transcription by binding to *WNT5A* promoter to form a DNA‐RNA triplex structure and recruit hnRNPA1 to increase its H3K4me3 levels.

### 3.7 *BCYRN1* activates Wnt/β‐catenin signaling pathway to promote the secretion of VEGF‐C

Given that WNT5A functions as the vital glycoprotein ligand triggering the activation of Wnt/β‐catenin signaling pathway,^32^ we performed the western blotting analysis to examine the alteration of crucial protein complexes involved in the Wnt/β‐catenin signaling pathway after silencing or overexpressing *BCYRN1*. As shown in Figures 5J–5K, the levels of WNT5A and β‐catenin were decreased and the level of phosphorylated glycogen synthase kinase 3 beta (GSK3β) was increased in *BCYRN1*‐silenced BCa cells. Conversely, overexpressing *BCYRN1* significantly upregulated the levels of WNT5A and β‐catenin and downregulated the level of phosphorylated GSK3β (Figures 5L and 5M). Furthermore, the TOP‐flash/FOP‐flash reporter assays revealed that β‐catenin signaling activity was significantly decreased in *BCYRN1*‐silenced BCa cells compared with the control, whereas it was increased after overexpressing *BCYRN1* (Figures S7K and S7L), suggesting that *BCYRN1* upregulates WNT5A to activate the Wnt/β‐catenin pathway in BCa cells.

The observation that *BCYRN1* significantly promoted the lymphangiogenesis and LN metastasis of BCa prompted us to explore the regulatory role of *BCYRN1* in the secretion of VEGF‐C which is the crucial inducer of tumor lymphangiogenesis.^6^ The results revealed that VEGF‐C secretion was markedly decreased by silencing *BCYRN1* expression, whereas it was increased after overexpressing *BCYRN1* (Figures 5N and 5O). We then evaluated whether the Wnt/β‐catenin signaling pathway was indispensable for *BCYRN1*‐promoted VEGF‐C secretion in BCa cells. *BCYRN1* overexpression significantly promoted VEGF‐C secretion, while the treatment with XAV939, a specific inhibitor for Wnt/β‐catenin pathway, reversed the stimulation of *BCYRN1*‐overexpressing on VEGF‐C secretion (Figures 5P and 5Q), confirming the essential function of Wnt/β‐catenin pathway in *BCYRN1*‐induced VEGF‐C secretion. Taken together, our results reveal that *BCYRN1* activates Wnt/β‐catenin signaling pathway to promote the secretion of VEGF‐C in BCa.

### 3.8 Exosomal *BCYRN1* is internalized by HLECs to promote lymphangiogenesis of BCa

**FIGURE 6:**
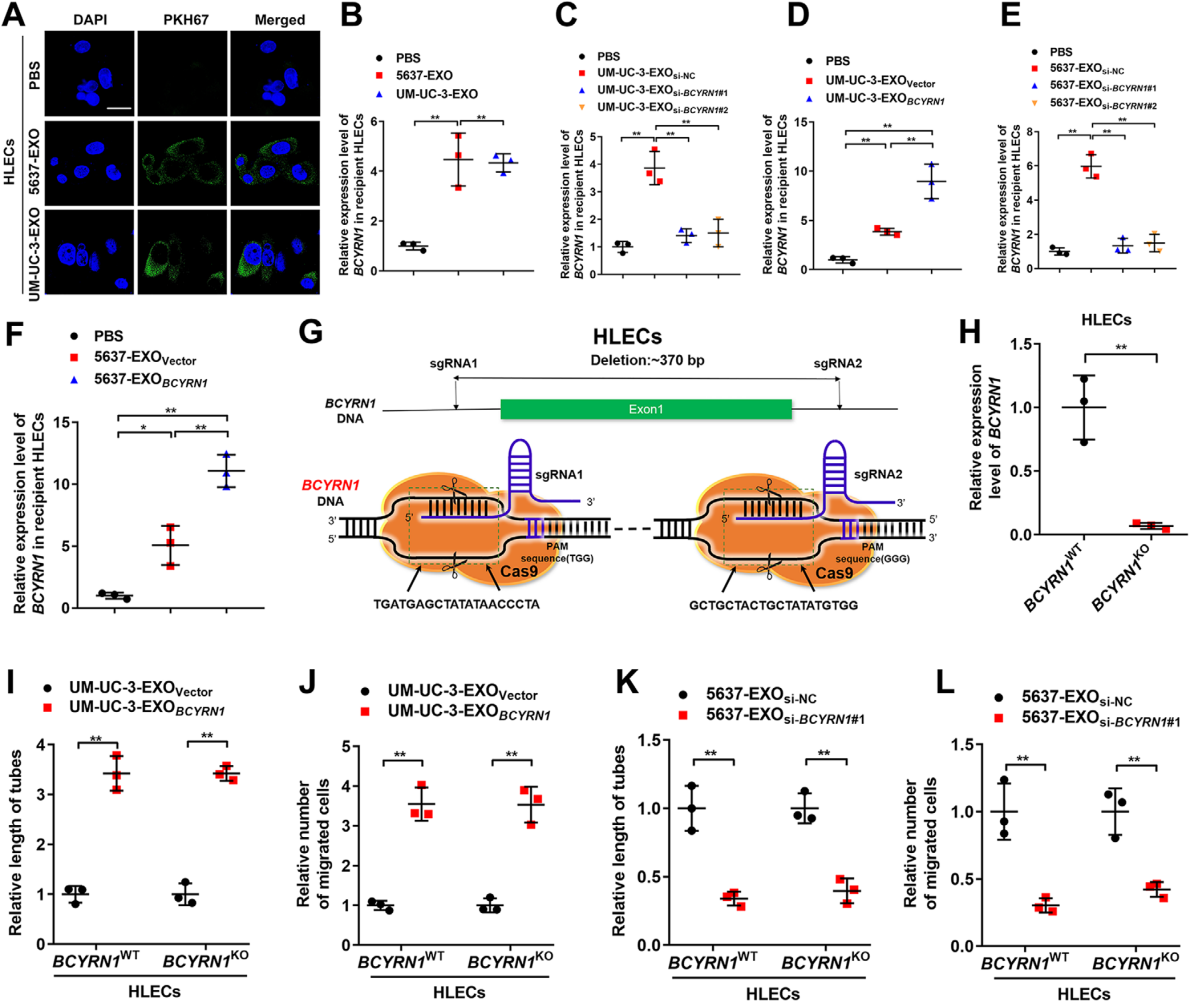
Exosomal *BCYRN1* is transmitted to HLECs to promote the lymphangiogenesis of BCa. (A) Representative fluorescence images of HLECs incubated with PKH‐67‐labeled exosomes from UM‐UC‐3 or 5637 cells. Scale bars: 5 μm. (B) qRT‐PCR analyzed *BCYRN1* expression in UM‐UC‐3‐EXO or 5637‐EXO‐treated HLECs. (C‐F) qRT‐PCR analysis of *BCYRN1* expression in HLECs after incubating with PBS and *BCYRN1*‐silenced or overexpressing BCa cells‐secreted exosomes. (G) Schematic diagram for the deletion of *BCYRN1* in HLECs using the CRISPR/Cas9 approach. (H) The knockout efficiency of *BCYRN1* in HLECs was assessed by qRT‐PCR analysis. (I‐L) Quantification of tube formation and Transwell migration of *BCYRN1* ^WT^ or *BCYRN1* ^KO^ HLECs treated *BCYRN1*‐overexpressing UM‐UC‐3 or *BCYRN1*‐silenced 5637 cell‐secreted exosomes. The statistical difference was assessed through one‐way ANOVA followed by Dunnett's tests in B‐F, and a two‐tailed Student's *t* test in H‐L. Error bars show the standard deviations derived from three independent experiments. *p < 0.05; **p < 0.01

Since our results showed that exosomal *BCYRN1* significantly promoted secretion of VEGF‐C and facilitated the lymphangiogenesis of BCa, we further examined the internalization of exosomal *BCYRN1* by HLECs. The isolated BCa cell‐secreted exosomes were labeled with PKH67 green dye and subjected to the incubation with HLECs, after which a punctate green fluorescence was observed in HLECs incubated with PKH67 green dye‐labeled BCa cell‐secreted exosomes, while no fluorescence was found in the PBS‐treated group (Figure 6A). Furthermore, our results showed that UM‐UC‐3 or 5637 cell‐secreted exosomes markedly upregulated the *BCYRN1* level in HLECs compared with that in the PBS‐treated group (Figure 6B). Incubation with exosomes secreted by *BCYRN1*‐overexpressing UM‐UC‐3 or 5637 cells (UM‐UC‐3‐EXO*_BCYRN1_* and 5637‐EXO*_BCYRN1_*) dramatically increased the level of *BCYRN1* in HLECs, while silencing *BCYRN1* abolished the upregulation of *BCYRN1* level in HLECs (Figures 6C–6F), indicating that BCa cell‐secreted exosomal *BCYRN1* is internalized by HLECs. Moreover, confocal microscopy analysis was conducted to reveal the significant enrichment of exosomes in LYVE1‐indicated lymphatic endothelial cells in the mice footpad primary tumor tissues intratumorally injected with PKH67‐labelled UM‐UC‐3‐EXO*_BCYRN1_* (Figure S8A), further confirming the uptake of exosomal *BCYRN1* by lymphatic endothelial cells *in vivo*.

To eliminate the possibility of BCa cell‐secreted exosomal *BCYRN1* inducing lymphangiogenesis by activating the transcription of endogenous *BCYRN1* in HLECs, the CRISPR‐Cas9 approach was performed to successfully establish *BCYRN1* knockout HLECs (Figures 6G and 6H). Consistently, the tube formation and migration of HLECs with *BCYRN1*‐KO or *BCYRN1*‐WT were both significantly promoted after treating with UM‐UC‐3‐EXO*_BCYRN1_* and 5637‐EXO*_BCYRN1_*, while silencing exosomal *BCYRN1* diminished their promoted effect on the tube formation and migration of HLECs (Figures 6I–6L and S8B‐S8E), indicating that BCa cell‐secreted exosomes induced lymphangiogenesis through transporting *BCYRN1* to HLECs instead of transcriptionally activating endogenous *BCYRN1* in HLECs. Collectively, our findings verify that exosomal *BCYRN1* is transported to HLECs to induce lymphangiogenesis of BCa.

### 3.9 Exosomal *BCYRN1* forms a feedforward loop with hnRNPA1/WNT5A/VEGFR3 regulatory axis

**FIGURE 7:**
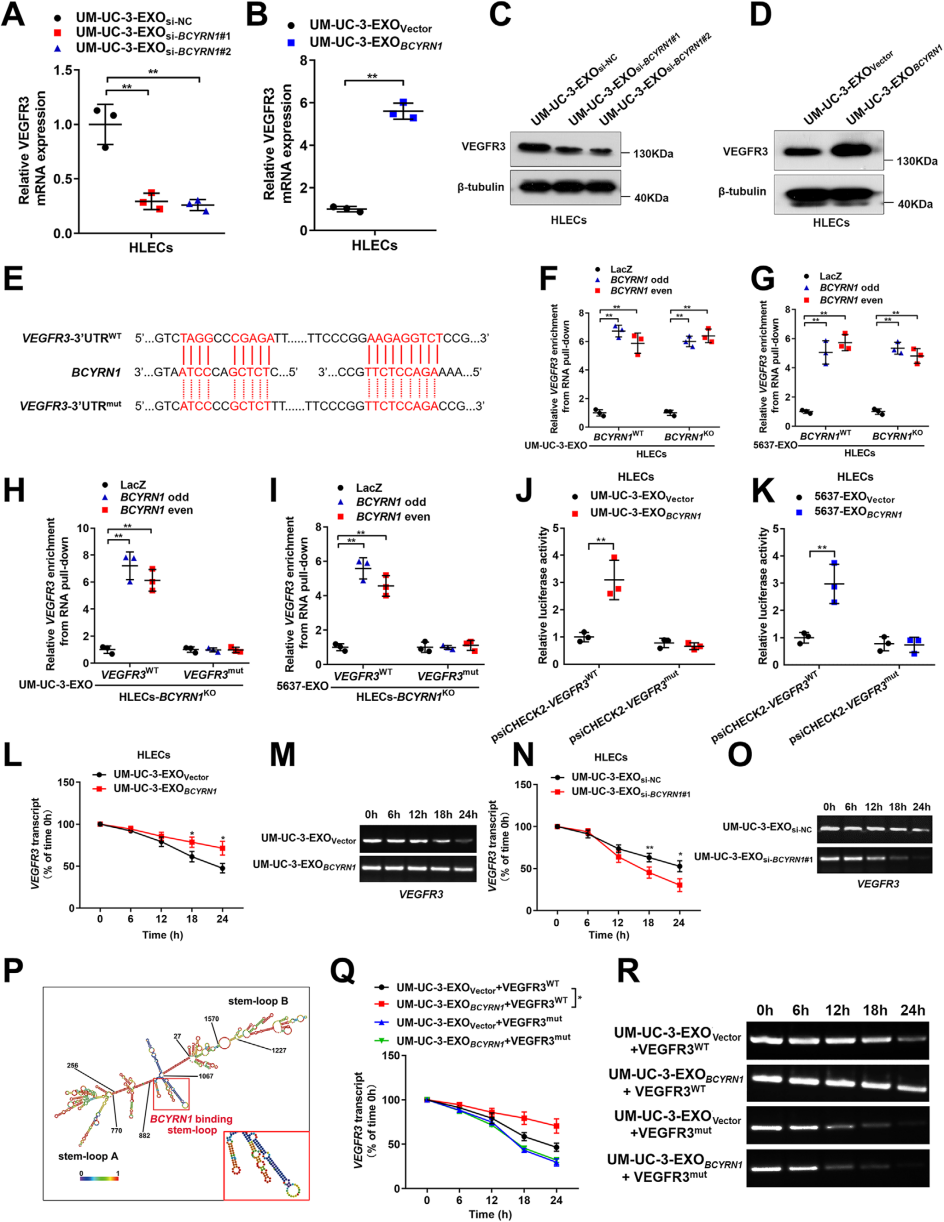
Exosomal *BCYRN1* upregulates VEGFR3 expression by enhancing its mRNA stability in HLECs. (A and B) qRT‐PCR analysis of VEGFR3 expression in HLECs treated with *BCYRN1*‐silenced or overexpressing UM‐UC‐3 cell‐secreted exosomes. (C and D) Western blotting analysis confirmed the upregulation of VEGFR3 in HLECs by exosomal *BCYRN1*. (E) Schematic sequences of potential binding sites between exosomal *BCYRN1* and the 3′‐UTR of VEGFR3. (F and G) RNA pull‐down assays with a biotin‐labeled *BCYRN1* probe showed the enrichment of the VEGFR3 3′‐UTR in *BCYRN1* ^WT^ or *BCYRN1* ^KO^ HLECs treated with UM‐UC‐3‐EXO or 5637‐EXO. (H and I) RNA pull‐down assays with a biotin‐labeled *BCYRN1* probe in *BCYRN1* ^KO^ HLECs after mutating the VEGFR3 3′‐UTR. (J and K) Luciferase assays in HLECs treated with exosomes secreted by *BCYRN1*‐overexpressing UM‐UC‐3 or 5637 cells with or without mutating the VEGFR3 3′‐UTR. (L‐O) Actinomycin D assays for VEGFR3 mRNA in HLECs treated with exosomes secreted from *BCYRN1*‐overexpressing or silenced UM‐UC‐3 cells. Quantification and representative images of agarose gel electrophoresis were shown. (P) The predicted stem‐loop structure of the *BCYRN1* binding regions in the VEGFR3 3′‐UTR was shown. (Q and R) Actinomycin D assays for VEGFR3 mRNA in UM‐UC‐3‐EXO_Vector_ or UM‐UC‐3‐EXO*_BCYRN1_*‐treated HLECs, with or without *BCYRN1*‐binding site mutations on the VEGFR3 3′‐UTR. Quantification and representative images of agarose gel electrophoresis were shown. The statistical difference was assessed through one‐way ANOVA followed by Dunnett's tests in A and F‐I; and a two‐tailed Student's *t* test in B, J, K, L, N, and Q. Error bars show the standard deviations derived from three independent experiments. *p < 0.05; **p < 0.01

It has been well‐characterized that VEGFR3 is widely involved in the sprout of lymphatic vasculature network and dissemination of tumors by initiating endothelial cell budding.^33^, ^34^ Our results showed that VEGFR3 expression was significantly upregulated in HLECs treated with UM‐UC‐3‐EXO*_BCYRN1_* and 5637‐EXO*_BCYRN1_*, but downregulated after incubating with exosomes secreted by *BCYRN1*‐silenced BCa cells (Figures 7A–7D and S8F‐S8I), suggesting that exosomal *BCYRN1* directly mediates VEGFR3 expression in HLECs.

Considering that specific interaction with the 3′‐untranslated regions (3′‐UTR) of target genes via the recognition of complementary sequences is the pivotal manner of exosomal lncRNA in regulating target genes,^35^, ^36^, ^37^ we used the Basic Local Alignment Search Tool to determine the possible complementary regions between *BCYRN1* and the 3′‐UTR of *VEGFR3* (Figure 7E). Moreover, RNA pull‐down assays with biotinylated oligonucleotides indicated the direct interaction between exosomal *BCYRN1* and the 3′‐UTR of *VEGFR3*, while inducing mutations of the potential binding sites in the 3′‐UTR of *VEGFR3* impaired its interaction with exosomal *BCYRN1* (Figures 7F–7I and S9A and S9B). Luciferase assays revealed that mutating the binding sites reduced the luciferase activity promoted by exosomal *BCYRN1* (Figures 7J and 7K), further confirming that these sites are essential for the interaction between exosomal *BCYRN1* and *VEGFR3*. Since the interaction with the 3′‐UTRs of mRNAs functions as a common characteristic contributed to RNA stability in cells, we further performed the actinomycin D assays to reveal that exosomes secreted by *BCYRN1*‐silenced BCa cells significantly shortened the half‐life of the *VEGFR3* mRNA, while exosomes from *BCYRN1*‐overexpressing BCa cells prolonged the half‐life of *VEGFR3* mRNA (Figures 7L‐7O and S9C‐S9F), indicating that exosomal *BCYRN1* promotes *VEGFR3* mRNA stability in HLECs. Moreover, RNAalifold was used to predict the secondary structure of exosomal *BCYRN1* binding sequences in the 3′‐UTR of *VEGFR3*, which form a stem‐loop structure (Figure 7P). Mutating the exosomal *BCYRN1* binding region in the 3′‐UTR of *VEGFR3* notably abolished the ability of exosomal *BCYRN1* to elongated the half‐life of *VEGFR3* mRNA (Figures 7Q and 7R and S9G and S9H). Collectively, our results demonstrate that exosomal *BCYRN1* promotes *VEGFR3* mRNA stability in HLECs via the interaction with the 3′‐UTR of *VEGFR3*, thus constituting a feedforward loop with hnRNPA1/WNT5A/VEGFR3 regulatory axis.

### 3.10 The feedforward loop‐induced VEGF‐C/VEGFR3 signaling is crucial for exosomal *BCYRN1*‐mediated LN metastasis

**FIGURE 8:**
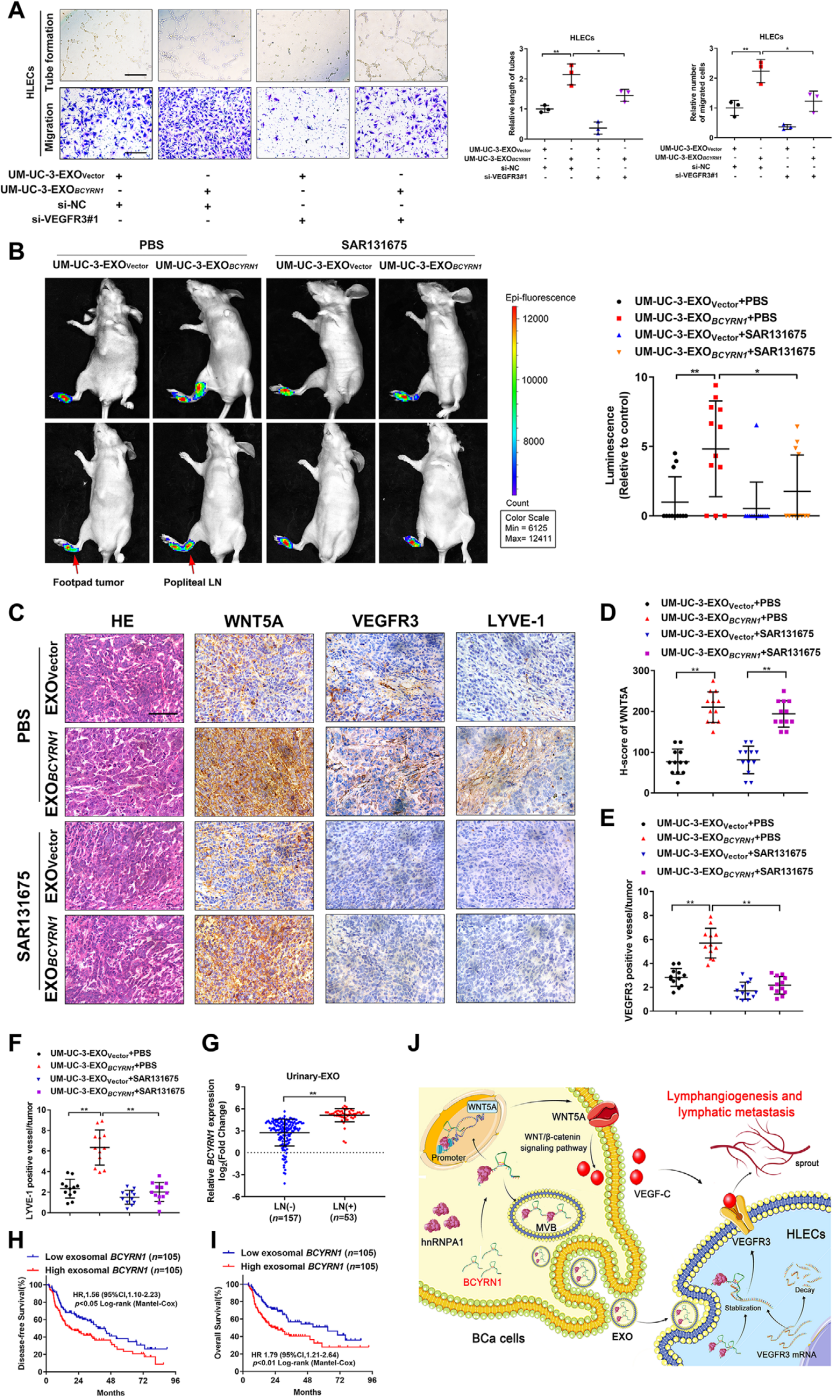
The feedforward loop‐induced VEGF‐C/VEGFR3 signaling is crucial for exosomal *BCYRN1*‐mediated LN metastasis of BCa. (A) Representative images and quantification of tube formation and Transwell migration for UM‐UC‐3‐EXO_Vector_ or UM‐UC‐3‐EXO*_BCYRN1_*‐treated HLECs transfected with si‐NC or si‐VEGFR3#1. Scale bars: 100 μm. (B) Representative images and quantification of bioluminescence for popliteal LN in UM‐UC‐3‐EXO_Vector_ or UM‐UC‐3‐EXO*_BCYRN1_*‐treated nude mice intratumorally injected with PBS or SAR131675 (*n* = 12). (C‐F) Representative images and histogram analysis of WNT5A expression and percentages of VEGFR3 or LYVE‐1‐indicated lymphatic vessel density in footpad primary tumor tissues of differently treated mice from the IHC analysis (*n* = 12). Scale bars: 50 μm. (G) qRT‐PCR analysis of *BCYRN1* expression in urinary‐EXO from patients with LN‐negative and LN‐positive BCa. (H and I) Kaplan–Meier survival analysis of the OS and DFS for patients with BCa with low versus high exosomal *BCYRN1* levels. The median exosomal *BCYRN1* level was used as the cutoff value. (J) Proposed model of exosomal *BCYRN1* promoting lymphangiogenesis and LN metastasis of BCa via the formation of a feedforward loop with hnRNPA1/WNT5A/VEGFR3 regulatory axis. The statistical difference was assessed through one‐way ANOVA followed by Dunnett's tests in A, B, and D‐F; and the nonparametric Mann–Whitney *U* test in G. Error bars show the standard deviations derived from three independent experiments. *p < 0.05; **p < 0.01

Next, we explored whether inhibiting the feedforward loop‐induced VEGF‐C/VEGFR3 signaling attenuated the promoted effect of exosomal *BCYRN1* in inducing lymphangiogenesis and LN metastasis of BCa. As shown in Figures 8A and S9I, incubation with exosomes secreted by *BCYRN1*‐overexpressing BCa cells promoted the tube formation and migration of HLECs, which was abolished by silencing *VEGFR3*, indicating that the upregulation of VEGFR3 expression in HLECs contributes to the exosomal *BCYRN1*‐mediated lymphangiogenesis of BCa *in vitro*. Moreover, we further determined the critical role of VEGFR3 in exosomal *BCYRN1*‐induced LN metastasis of BCa *in vivo* by blocking VEGFR3 activity with its specific inhibitor, SAR131675, in the constructed footpad‐popliteal LN metastasis model. Strikingly, the exosomal *BCYRN1*‐induced promotion of the fluorescence intensity in nude mice popliteal LN was significantly impaired by treating with SAR131675, as determined using IVIS (Figure 8B). The popliteal LN metastasis rate in UM‐UC‐3‐EXO*_BCYRN1_* +SAR131675 group was significantly lower than that in UM‐UC‐3‐EXO*_BCYRN1_+*PBS group (Table S6). Furthermore, exosomal *BCYRN1* significantly increased the WNT5A expression and the density of VEGR3 or LYVE‐1‐indicated lymphatic vessels in the footpad primary tumor tissues while treating with SAR131675 markedly attenuated the exosomal *BCYRN1*‐induced increase of lymphatic vessels (Figures 8C–8F), suggesting that blocking VEGFR3 inhibits exosomal *BCYRN1*‐mediated lymphangiogenesis of BCa *in vivo*. Taken together, these findings demonstrate that the feedforward loop‐induced VEGF‐C/VEGFR3 signaling is indispensable for exosomal *BCYRN1*‐mediated lymphangiogenesis and LN metastasis of BCa.

### 3.11 Exosomal *BCYRN1* correlates with LN metastasis of BCa patients

Urinary exosomal lncRNA is considered as encouraging diagnosis biomarker for patients with BCa.^38^ Therefore, the exploration of the clinical association of urinary exosomal *BCYRN1* with BCa LN metastasis is of great importance. We isolated the urinary‐EXOs from 210 BCa patients in our cohort and conducted qRT‐PCR analysis to reveal that the urinary exosomal *BCYRN1* levels were markedly higher in patents with LN metastatic BCa than that in patients without LN metastasis (Figure 8G). Exosomal *BCYRN1* overexpression positively correlated with higher tumor stage and lymphatic metastasis of patients with BCa (Table S1). Moreover, a higher level of urinary exosomal *BCYRN1* was accompanied with poor disease‐free survival and overall survival (OS) of patients with BCa, as confirmed by Kaplan–Meier analysis (Figures 8H and 8I). Statistical analysis further determined the vital role of exosomal *BCYRN1* as a prognostic factor for patients with BCa (Table S7 and Table S8), indicating the significant clinical relevance of exosomal *BCYRN1* in BCa and supporting exosomal *BCYRN1* as a promising therapeutic target for patients with LN metastatic BCa (Figure 8J).

## 4 DISCUSSION

Lymphatic metastasis is the leading cause for the poor prognosis of BCa patients.^39^ Emerging evidence indicated that TME induced by lymphangiogenic signal transduction drives LN metastasis of tumors,^40^, ^41^ but its underlying regulatory mechanisms in lymphatic metastasis of BCa are still unclear. In the present study, we have verified for the first time that lncRNA *BCYRN1* was overexpressed in the urinary‐EXO from BCa patients and promoted the VEGF‐C‐dependent lymphangiogenesis and LN metastasis of BCa. *BCYRN1* epigenetically upregulated WNT5A expression to activate Wnt/β‐catenin pathway and facilitated the secretion of VEGF‐C in BCa cells. Moreover, exosomal *BCYRN1* was delivered to HLECs to enhance VEGFR3 expression and constituted a feedforward loop with hnRNPA1/WNT5A/VEGFR3 regulatory axis resulting in LN metastasis of BCa. The present study systematically elucidates the precise mechanism by which exosomal *BCYRN1* induces lymphatic metastasis of BCa, supporting that directly targeting *BCYRN1* is an encouraging intervention for the treatment of BCa patients with LN metastasis.

VEGF‐C/VEGFR3 signaling is essential for tumor LN metastasis by promoting the lymphangiogenesis which provides a potential entrance channel for tumor cells to the lymphatic drainage system.^8^ The deletion of VEGF‐C aborts the proliferation and migration ability of nascent lymphatic endothelial cells, inhibiting lymphatic metastasis of tumors.^7^ Nevertheless, the regulatory mechanisms in activating the VEGF‐C/VEGFR3 signaling triggering LN metastasis of BCa remain largely unknown. Herein, our results demonstrated the synergistic mechanism of exosomal *BCYRN1* in the stimulation of VEGF‐C/VEGFR3 signaling in BCa. We revealed that *BCYRN1*‐mediated activation of Wnt/β‐catenin signaling pathway to induce the secretion of VEGF‐C. Furthermore, *BCYRN1* played a crucial regulatory role in upregulating the expression of VEGFR3 via exosomal transmission, thus coordinately mediating VEGF‐C/VEGFR3 signaling‐induced lymphangigenesis and LN metastasis of BCa. Our results fully elaborate the molecular mechanism in exosomal *BCYRN1*‐induced synergistical activation of VEGF‐C/VEGFR3 signaling, deepening our knowledge in the mediation of VEGF‐C‐dependent lymphatic metastasis of tumor.

Previous study reported that *BCYRN1* was an oncogenic lncRNA contributing to the progression of various cancers by serving as a crucial microRNA sponge.^42^ Overexpression of *BCYRN1* competitively bound with miR‐204‐3p to regulate the tumorigenesis of colorectal cancer.^43^ However, the biological role of *BCYRN1* in exosome‐induced LN metastasis of BCa remains unclear. Herein, we revealed that *BCYRN1* played a vital role in exosome‐mediated communication between BCa cells and HLECs and triggered the lymphangiogenesis of BCa. *BCYRN1* was significantly overexpressed in exosomes secreted by BCa cells and was transported to HLECs to stabilize VEGFR3 mRNA by interacting with its 3′‐UTR, thus synergistically activating the VEGF‐C/VEGFR3 signaling in BCa. Our results highlight a novel regulatory role of *BCYRN1* in exosome‐mediated VEGF‐C/VEGFR3 signaling‐induced LN metastasis of BCa and support *BCYRN1* as a therapeutic target for BCa patients.

Feedforward loop is a critical manner for the regulation of transcription networks during tumor progression.^44^, ^45^ It is proposed that the formation of a feedforward loop enables the oncogenic signaling to rapidly drive transcriptional program, leading to the metastasis of tumors.^46^, ^47^, ^48^ However, the biological role and precise mechanisms of feedforward loop in remodeling TME driving tumor LN metastasis have not been fully elucidated. Our present study demonstrated that the feedforward loop was formed between exosomal *BCYRN1* and hnRNPA1/WNT5A/VEGFR3 regulatory axis to reinforce the VEGF‐C/VEGFR3 signaling in TME and enhance VEGF‐C‐dependent lymphangiogenesis and LN metastasis of BCa. Importantly, blocking the feedforward loop‐induced VEGF‐C/VEGFR3 signaling with its specific inhibitor SAR131675 inhibited exosomal *BCYRN1*‐mediated LN metastasis *in vivo*, suggesting that the formation of this loop functions as a detector to enable the rapid and effective response for the activation of VEGF‐C/VEGFR3 signaling and supports the promotion of BCa LN metastasis. These results reveal a novel mechanism in the induction of lymphangiogenesis through exosomal lncRNA‐mediated feedforward loop, providing a new perspective of feedforward loop in mediating the lymphatic metastasis of tumors.

Exosomes served as a nanometer biological microvesicle in transmitting various biomolecule have been characterized as a promising therapeutic target for human diseases.^49^, ^50^, ^51^ Relevant studies have demonstrated the effectiveness achieved by targeting exosomal lncRNA to improve the immunotherapy and treat the metastatic peritoneal cancer, suggesting the wide application prospects of exosomal lncRNA in the treatment of tumors.^52^ Here, we revealed that high level of *BCYRN1* in urinary‐EXO from BCa patients was associated with the LN metastasis and poor prognosis of patients, providing the clinical evidence for targeting exosomal *BCYRN1* to treat LN metastatic BCa. Recently, oligonucleotides targeting the oncogenic lncRNA have realized an observable anti‐tumor effectiveness *in vivo* through selectively modulating the pathological alteration of lncRNA in tumor cells.^53^ Our results showed that silencing exosomal *BCYRN1* prominently suppressed the VEGF‐C/VEGFR3 signaling‐induced lymphangiogenesis and LN metastasis of BCa *in vitro* and *in vivo*, indicating that inhibiting exosomal *BCYRN1* with specific‐designed targeting oligonucleotide may become a promising therapeutic strategy to block the pathological overactivation of VEGF‐C/VEGFR3 signaling and develop a promising intervention for patients with LN metastatic BCa.

## 5 CONCLUSIONS

In summary, our findings highlight the crucial role of exosomal *BCYRN1* in the lymphangiogenesis and LN metastasis of BCa by forming a feedforward loop with hnRNPA1/WNT5A/VEGFR3 regulatory axis. Systematically exploring the molecular mechanism of exosomal *BCYRN1* in mediating LN metastasis of BCa via a VEGF‐C‐dependent manner provides a new insight into exosomal lncRNA‐induced lymphatic metastasis of tumors. Our findings support that targeting exosomal *BCYRN1* to block the pathological overactivation of VEGF‐C/VEGFR3 signaling is a promising clinical intervention for patients with LN metastatic BCa.

## CONFLICT OF INTEREST

The authors have declared that no conflict of interest exists.

## ETHICS APPROVAL AND CONSENT TO PARTICIPATE

We obtained the informed consent of involved patients and the ethical approval of the Committees for Ethical Review of Research Involving Human Subjects at Sun Yat‐sen University (approval number:2013[61]).

## AUTHOR CONTRIBUTIONS

Hanhao Zheng, Changhao Chen, and Tianxin Lin contributed to the design of study. Min Yu, Yao Kong, and Bowen Gao performed the clinical statistical analysis. Mingjie An, Yiyao Ya, and Jian Huang conducted the CHIRP and ChIP assays. Yuting Li, Wang He and Yan Lin carried out the ISH and IHC analysis. Keji Xie, Hanhao Zheng, and Changhao Chen conducted the in vitro experiments. YuMing Luo performed the in vivo assays. Hanhao Zheng, Changhao Chen, and Tianxin Lin wrote the manuscript. All the authors have read and approved the final manuscript.

## AVAILABILITY OF DATA AND MATERIALS

The data of next‐generation sequencing in our study (GSE156308) are obtainable in a public repository from NCBI (https://www.ncbi.nlm.nih.gov/geo/query/acc.cgi). All relevant data in the present study are obtainable from the authors.

## Supporting information

Supporting Information
