# Supplementary material for: Tumor‐derived exosomal BCYRN1 activates WNT5A/VEGF‐C/VEGFR3 feedforward loop to drive lymphatic metastasis of bladder cancer
Source: Clin Transl Med. 2021 Jul 19;11(7):e497. doi: 10.1002/ctm2.497 (PMC8288020; doi:10.1002/ctm2.497)
Supplement: Supplementary file 1 — Supporting Information [file CTM2-11-e497-s001.doc]

**Tumor-derived exosomal *BCYRN1* activates WNT5A/ VEGF-C/VEGFR3 feedforward loop to drive lymphatic metastasis of bladder cancer**

Hanhao Zheng^1,2#^, Changhao Chen^1,2#*^, Yuming Luo^3#^, Min Yu^3#^, Wang He^1,2^, Mingjie An^1,2^, Bowen Gao^4^, Yao Kong^3^, Yiyao Ya^5^, Yan Lin^1,2^, Yuting Li^3^, Keji Xie^5*^, Jian Huang^1,2*^, Tianxin Lin^1,2*^

^1^Department of Urology, Sun Yat-sen Memorial Hospital, Guangzhou, Guangdong, P. R. China

^2^Guangdong Provincial Key Laboratory of Malignant Tumor Epigenetics and Gene Regulation, Sun Yat-sen Memorial Hospital, State Key Laboratory of Oncology in South China, Guangzhou, Guangdong, P. R. China

^3^Department of General Surgery, Guangdong Provincial People’s Hospital, Guangdong Academy of Medical Sciences, Guangzhou, Guangdong, P. R. China

^4^Department of Pancreatobiliary Surgery, Sun Yat-sen Memorial Hospital, Guangzhou, Guangdong, P. R. China

^5^Department of Urology, Guangzhou First People’s Hospital, School of Medicine, South China University of Technology, Guangzhou, China

* Correspondence to:

Tianxin Lin & Jian Huang & Changhao Chen. Department of Urology, Sun Yat-sen Memorial Hospital, 107 Yanjiangxi Road, Yuexiu District, Guangzhou, Guangdong Province, P. R. China, postal code: 510120; E-mail: [lintx@mail.sysu.edu.cn](mailto:lintx@mail.sysu.edu.cn) & huangj8@mail.sysu.edu.cn & chenchh53@mail.sysu.edu.cn; Tel.: +86-13724008338, Work Tel.: +86-20-34070447; Fax: +86-20-81332336.

Keji Xie. Department of Urology, Guangzhou First People’s Hospital, School of Medicine, South China University of Technology, Guangzhou, China, postal code: 510180; E-mail: xiekeji@sina.com; Tel.: +86-20-34070447; Fax: +86-20-81332336.

^#^These authors contributed equally to this study.

**Supplemental Figures**

**
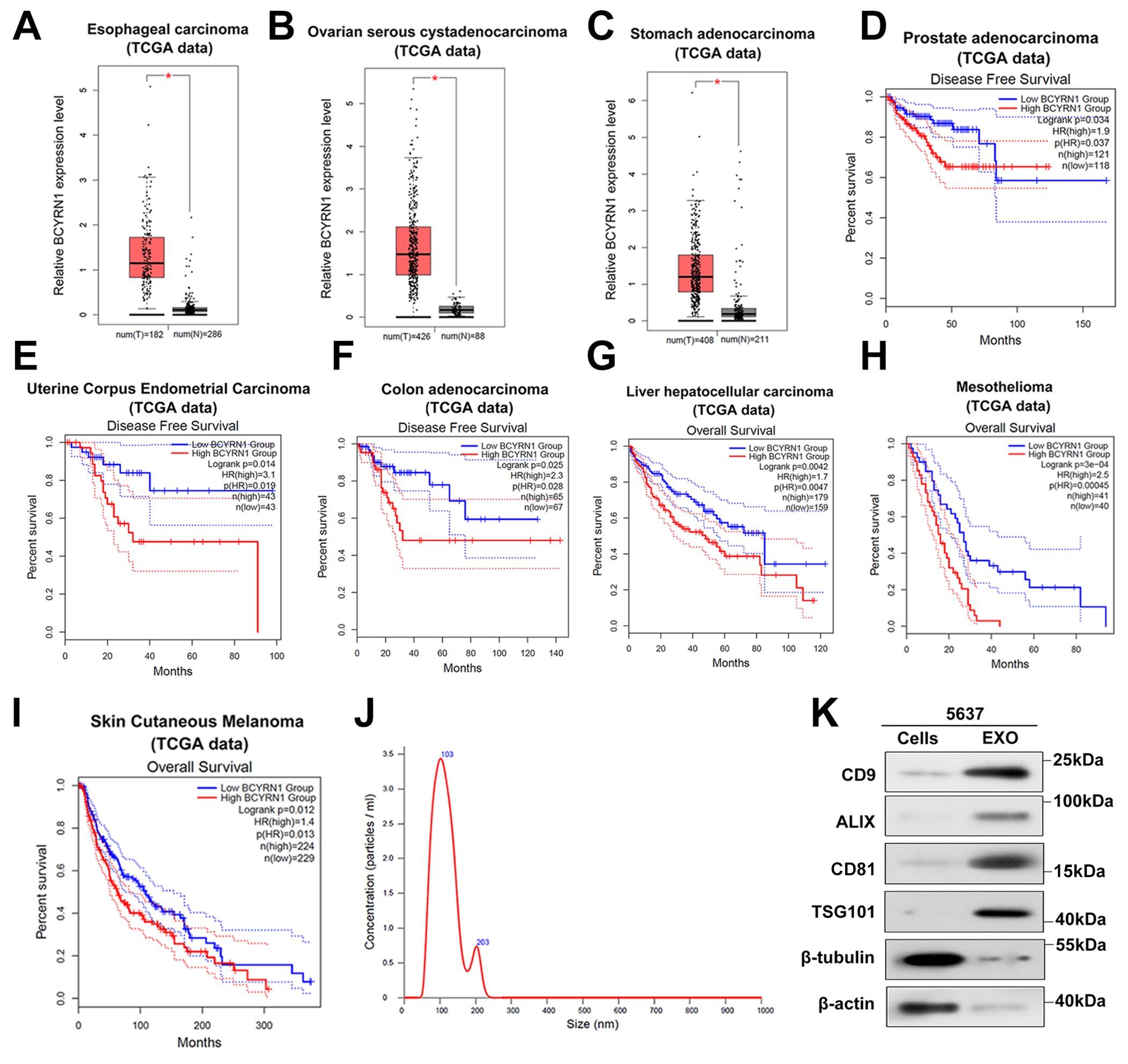
FIGURE S1 *BCYRN1* is overexpressed in human cancers. (A-C)** The *BCYRN1* overexpression in multiple types of cancers was revealed through the analysis of TCGA database. **(D-I)** Kaplan-Meier survival analysis of the DFS and OS for different types of cancer patients with low vs. high *BCYRN1* expression. The cutoff value is the best cutoff. **(J)** NTA identified the characteristic of exosomes isolated from 5637 cells culture media. **(K)** Western blotting analysis of the indicated exosomal markers in 5637 cells lysate and purified exosomes from culture media. The statistical difference was assessed through Nonparametric Mann-Whitney *U* test in **A-C**. Error bars showed the standard deviations derived from three independent experiments. **p* < 0.05; ***p* < 0.01.

**
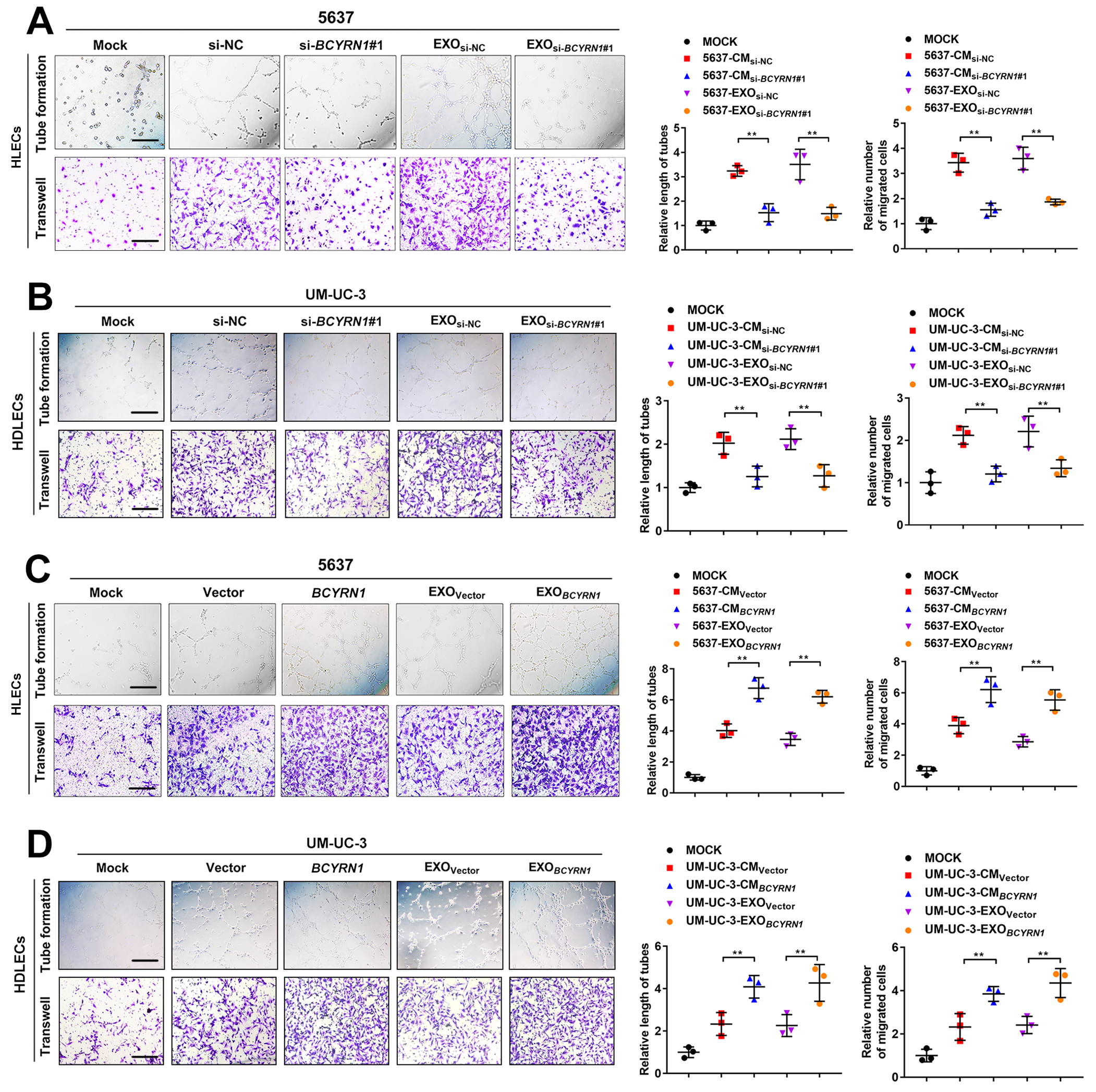
FIGURE S2 Exosomal *BCYRN1* promotes the lymphangiogenesis of BCa *in vitro*. (A)** Representative images and quantification of tube formation and Transwell migration for HLECs treated with PBS, culture media from 5637_si-NC_ or 5637_si-_*_BCYRN1_*_#1_ cells and their corresponding exosomes. Scale bars: 100 μm. **(B)** Representative images and quantification of tube formation and Transwell migration for HDLECs treated with PBS, culture media from UM-UC-3_si-NC_ or UM-UC-3_si-_*_BCYRN1_*_#1_ cells, and their corresponding exosomes. Scale bars: 100 μm. **(C)** Representative images and quantification of tube formation and Transwell migration for HLECs treated with PBS, culture media from 5637_Vector_ or 5637*_BCYRN1_*cells and their corresponding exosomes. Scale bars: 100 μm. **(D)** Representative images and quantification of tube formation and Transwell migration for HDLECs treated with PBS, culture media from UM-UC-3_Vector_ or UM-UC-3*_BCYRN1_* cells, and their corresponding exosomes. Scale bars: 100 μm. The statistical difference was assessed through one-way ANOVA followed by Dunnett’s tests in **A-D**. Error bars showed the standard deviations derived from three independent experiments. **p* < 0.05; ***p* < 0.01.

**
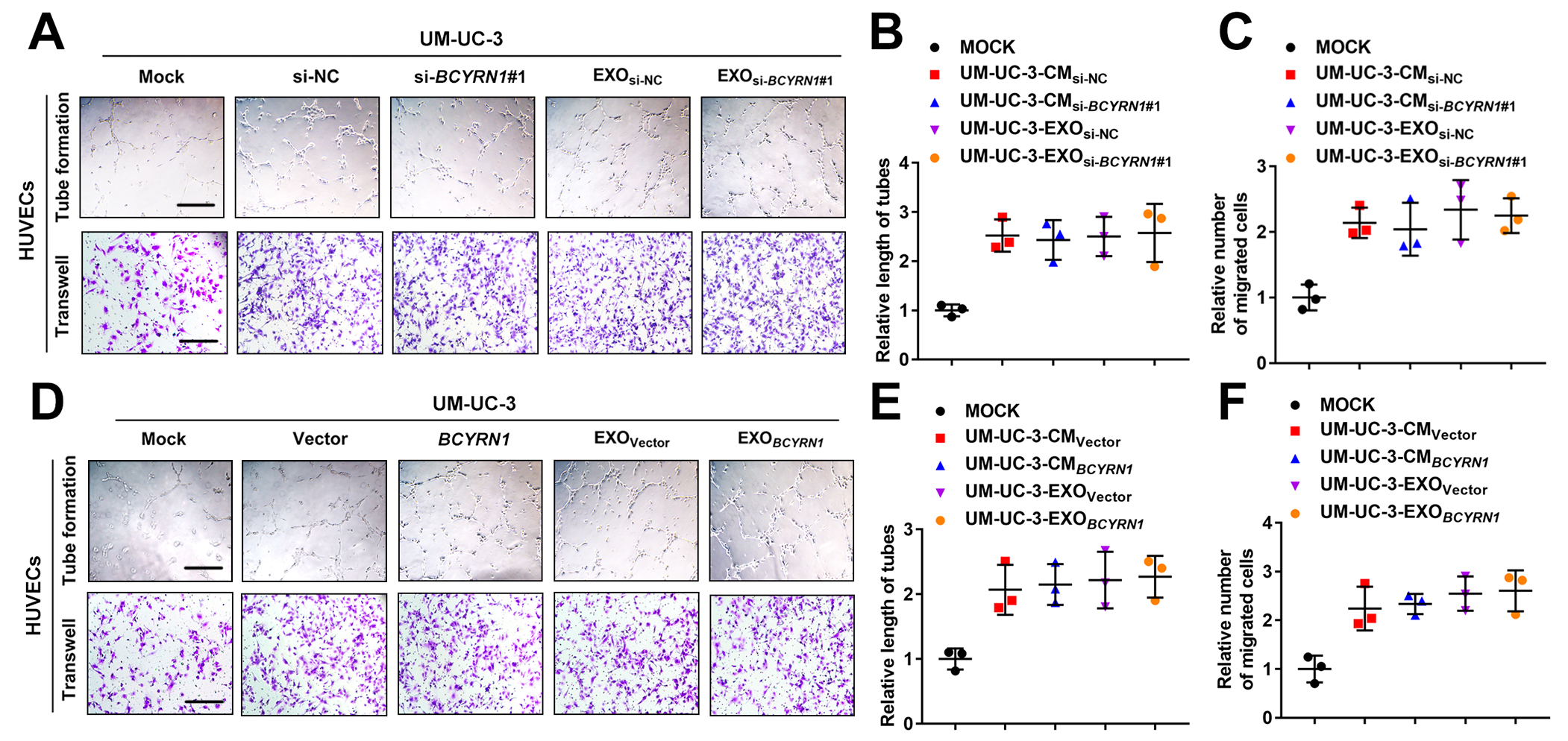
FIGURE S3 Exosomal *BCYRN1* has no significant effect in the angiogenesis of BCa *in vitro*. (A-C)** Representative images and quantification of tube formation and Transwell migration for HUVECs treated with PBS, culture media from UM-UC-3_si-NC_ or UM-UC-3_si-_*_BCYRN1_*_#1_ cells, and their corresponding exosomes. Scale bars: 100 μm. **(D-E)** Representative images and quantification of tube formation and Transwell migration for HUVECs treated with PBS, culture media from UM-UC-3_Vector_ or UM-UC-3*_BCYRN1_* cells, and their corresponding exosomes. Scale bars: 100 μm. The statistical difference was assessed through one-way ANOVA followed by Dunnett’s tests in **B, C, E** and **F.** Error bars show the standard deviations derived from three independent experiments.**p* < 0.05; ***p* < 0.01.


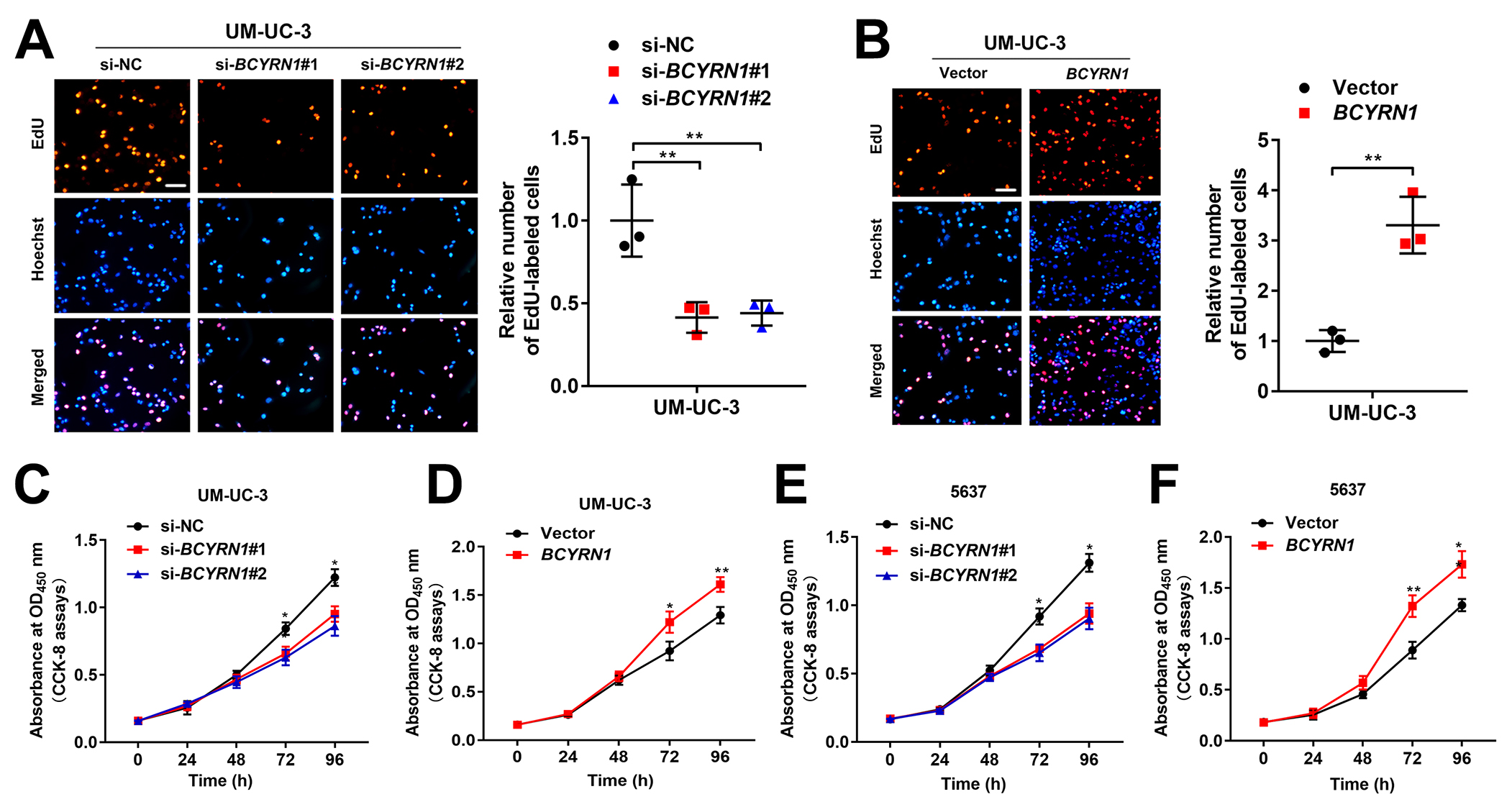


**FIGURE S4 *BCYRN1* promotes the proliferation of BCa *in vitro*. (A and B)** Representative images and quantification of EdU assays after silencing or overexpressing *BCYRN1* in UM-UC-3 cells. Scale bars: 100 μm. **(C-F)** Cell viability was assessed by CCK-8 assays after silencing or overexpressing *BCYRN1* in UM-UC-3 or 5637 cells. The statistical difference was assessed through one-way ANOVA followed by Dunnett’s tests in **A**, **C** and **E,** and two-tailed Student’s t test in **B**, **D** and **F.** Error bars show the standard deviations derived from three independent experiments. **p* < 0.05; ***p* < 0.01.

**
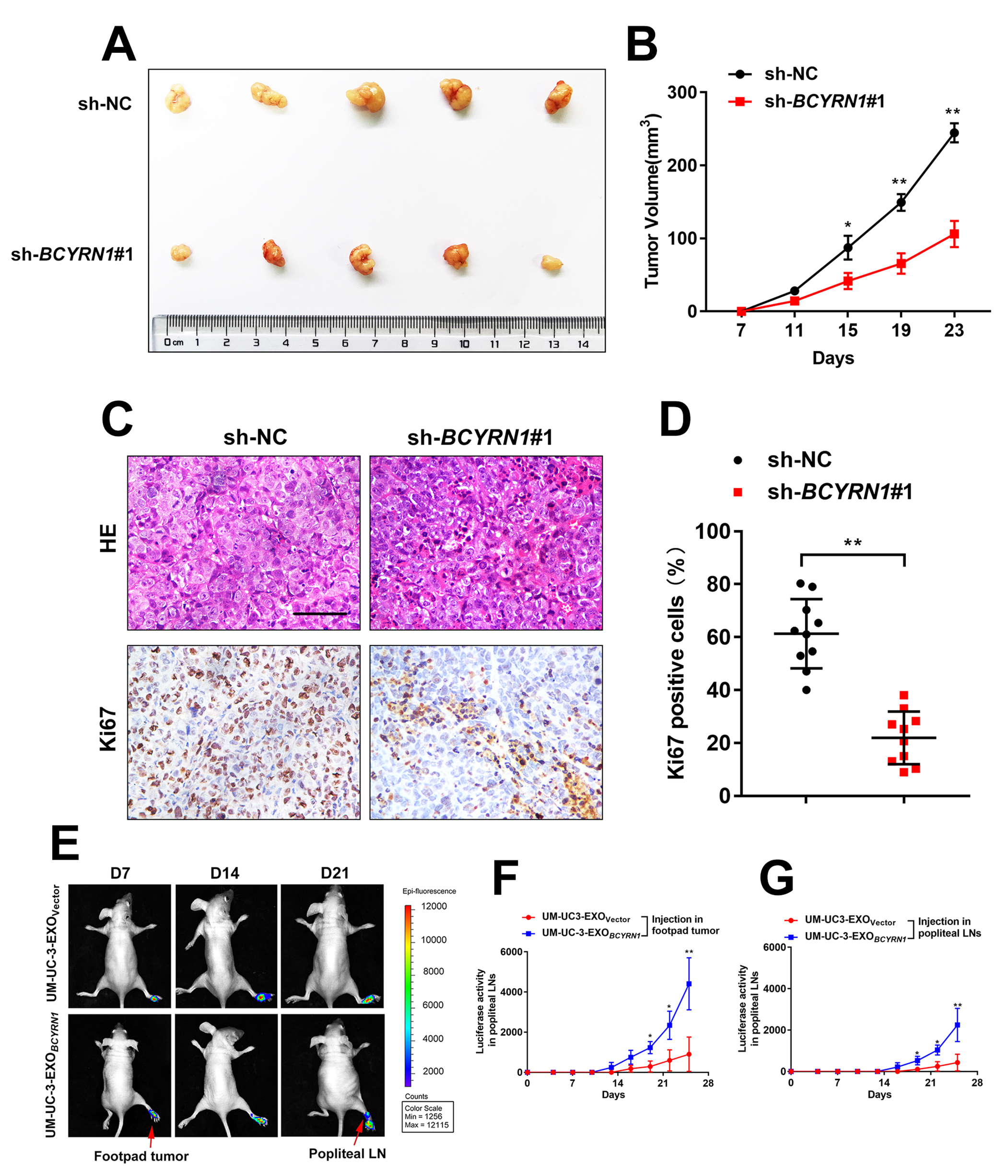
FIGURE S5 *BCYRN1* promotes the tumorigenicity and LN metastasis of BCa *in vivo*. (A)** Representative images of subcutaneous xenograft tumors from mice of sh-NC or sh-*BCYRN1*#1 group (n=10). **(B)** The tumor volumes in sh-NC or sh-*BCYRN1*#1 group were measured. **(C and D)** Representative images and quantification of Ki-67 expression in tumor tissues from mice of sh-NC or sh-*BCYRN1*#1 group. Scale bars: 50 μm. (**E and F)** Representative images and quantification of bioluminescence for popliteal LN in the nude mice model intratumorally injected with UM-UC-3-EXO_Vector_ or UM-UC-3-EXO*_BCYRN1_* were recorded across time (n = 12). (**G)** Quantification of bioluminescence for popliteal LN in the nude mice model injected with UM-UC-3-EXO_Vector_ or UM-UC-3-EXO*_BCYRN1_* in the popliteal LNs were recorded across time (n = 12). The statistical difference was assessed through two-tailed Student’s t test in **B**, **D**, **F** and **G**. Error bars show the standard deviations derived from three independent experiments. **p* < 0.05; ***p* < 0.01.

**
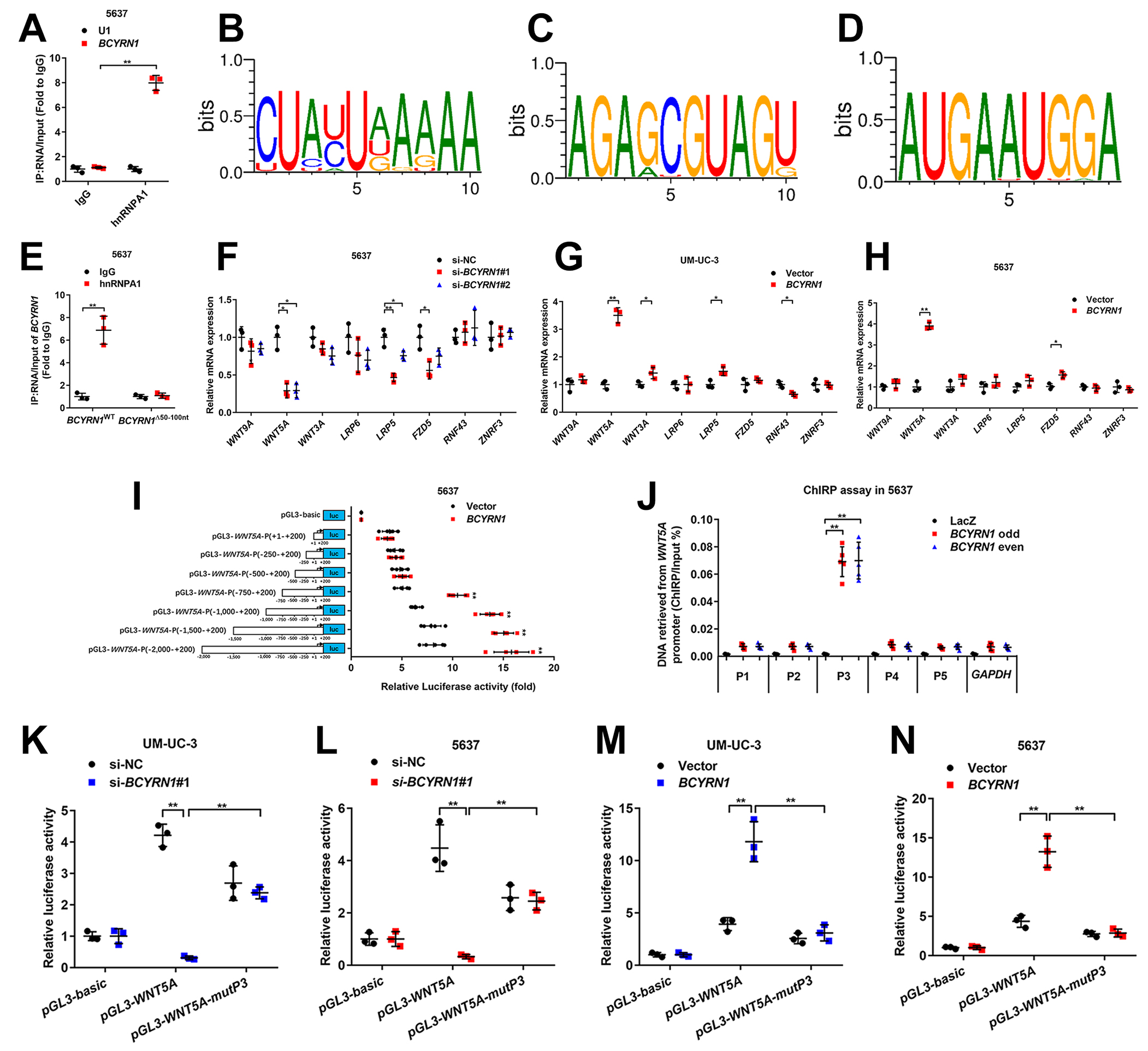
FIGURE S6 *BCYRN1* activates WNT5A transcription by interacting with its promoter. (A)** The enrichment of *BCYRN1* by anti-hnRNPA1 antibody after RIP assays confirmed the direct interaction of *BCYRN1* with hnRNPA1 in 5637 cells. IgG was used as negative control and U1 as non-specific control. **(B-D)** The potential binding sequence motifs of hnRNPA1 were predicted by POSTAR2 through the sequence motif finding tool HOMER, and **B** was identified as the most possible binding sequence motif of hnRNPA1 in *BCYRN1* through the recognition of matching sequences. **(E)** qRT-PCR analysis of RIP assays after deleting the 50-100 nt regions of *BCYRN1* in 5637 cells. **(F)** Core genes involved in the Wnt/β-catenin pathway were detected using qRT-PCR analysis in *BCYRN1*‑silenced 5637 cells. **(G and H)** Related genes associated with Wnt/β-catenin pathway were detected by qRT-PCR analysis in *BCYRN1*-overexpressing UM-UC-3 and 5637 cells. **(I)** Relative luciferase activity was detected in *BCYRN1*-overexpressing 5637 cells transfected with truncate *WNT5A* promoter plasmids. **(J)** ChIRP assays detected the *BCYRN1*-associated chromatin in 5637 cells. **(K-N)** Luciferase assays in *BCYRN1*-silenced or overexpressing BCa cells transfected with WT or *BCYRN1* binding regions mutated *WNT5A* promoter plasmids. The statistical difference was assessed through Two-tailed Student’s *t* test in **A, E, G** and **H**, one-way ANOVA followed by Dunnett’s tests in **F** and **I-N**. Error bars showed the standard deviations derived from three independent experiments. **p* < 0.05; ***p* < 0.01.

**
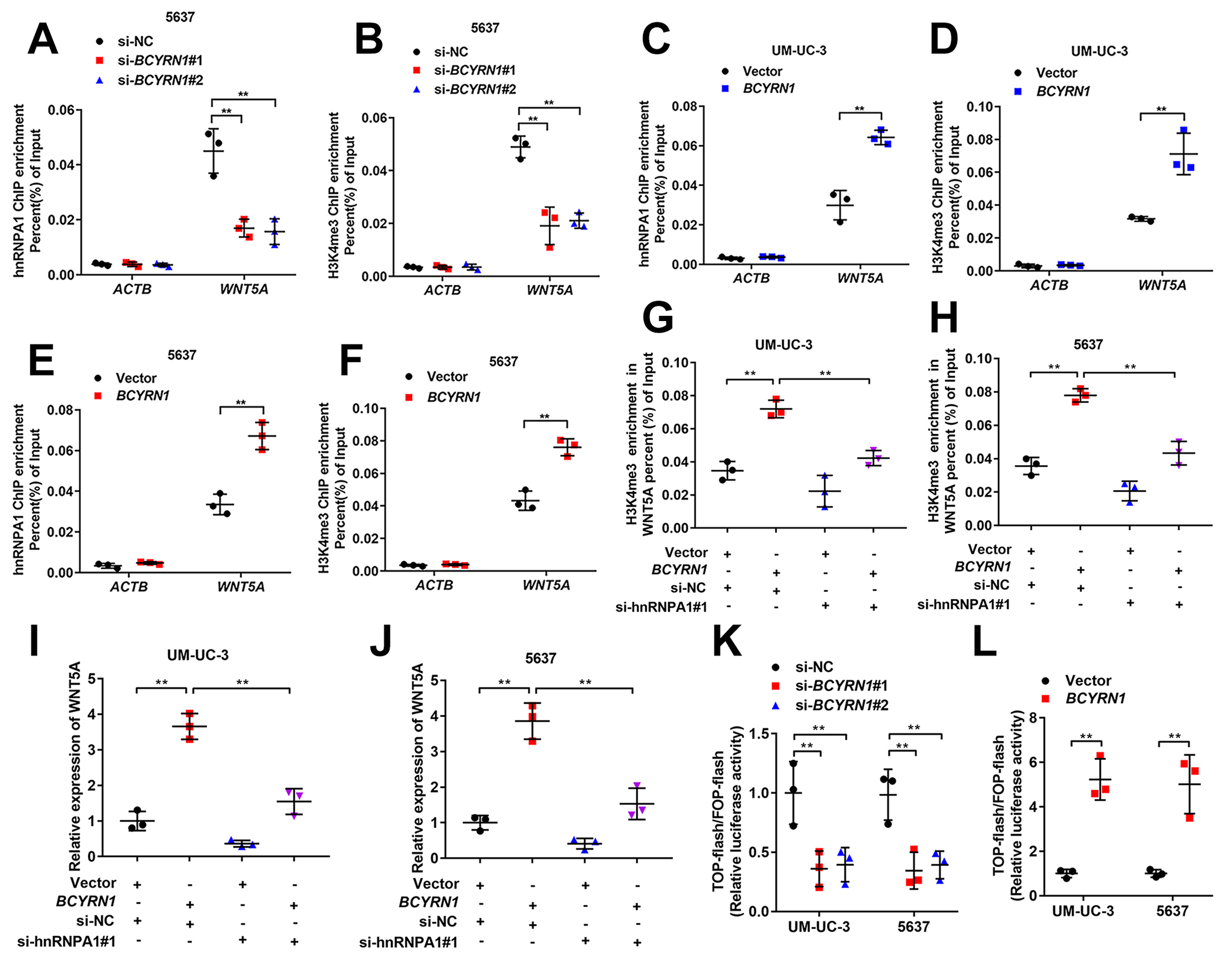
FIGURE S7 *BCYRN1* upregulates WNT5A expression by inducing hnRNPA1-associated H3K4me3 modification on WNT5A promoter.** **(A-F)** ChIP assays evaluated the hnRNPA1 occupancy and H3K4me3 status in *WNT5A* promoter in *BCYRN1*-silenced 5637 cells and *BCYRN1*-overexpressing UM-UC-3 and 5637 cells. **(G and H)** ChIP assays evaluated the H3K4me3 status in *WNT5A* promoter in *BCYRN1*-overexpressing BCa cells transfected with si-NC or si‑hnRNPA1#1. **(I and J)** qRT-PCR analysis revealed that silencing hnRNPA1 impaired the *BYCRN1*-induced upregulation of WNT5A in UM-UC-3 or 5637 cells. **(K and L)** The TOP-flash/FOP-flash luciferase activity was measured after silencing or overexpressing *BCYRN1* in UM-UC-3 and 5637 cells. The statistical difference was assessed through one-way ANOVA followed by Dunnett’s tests in **A**, **B** and **G-K**, and Two-tailed Student’s *t* test in **C-F** and **L**. Error bars showed the standard deviations derived from three independent experiments. **p* < 0.05; ***p* < 0.01.


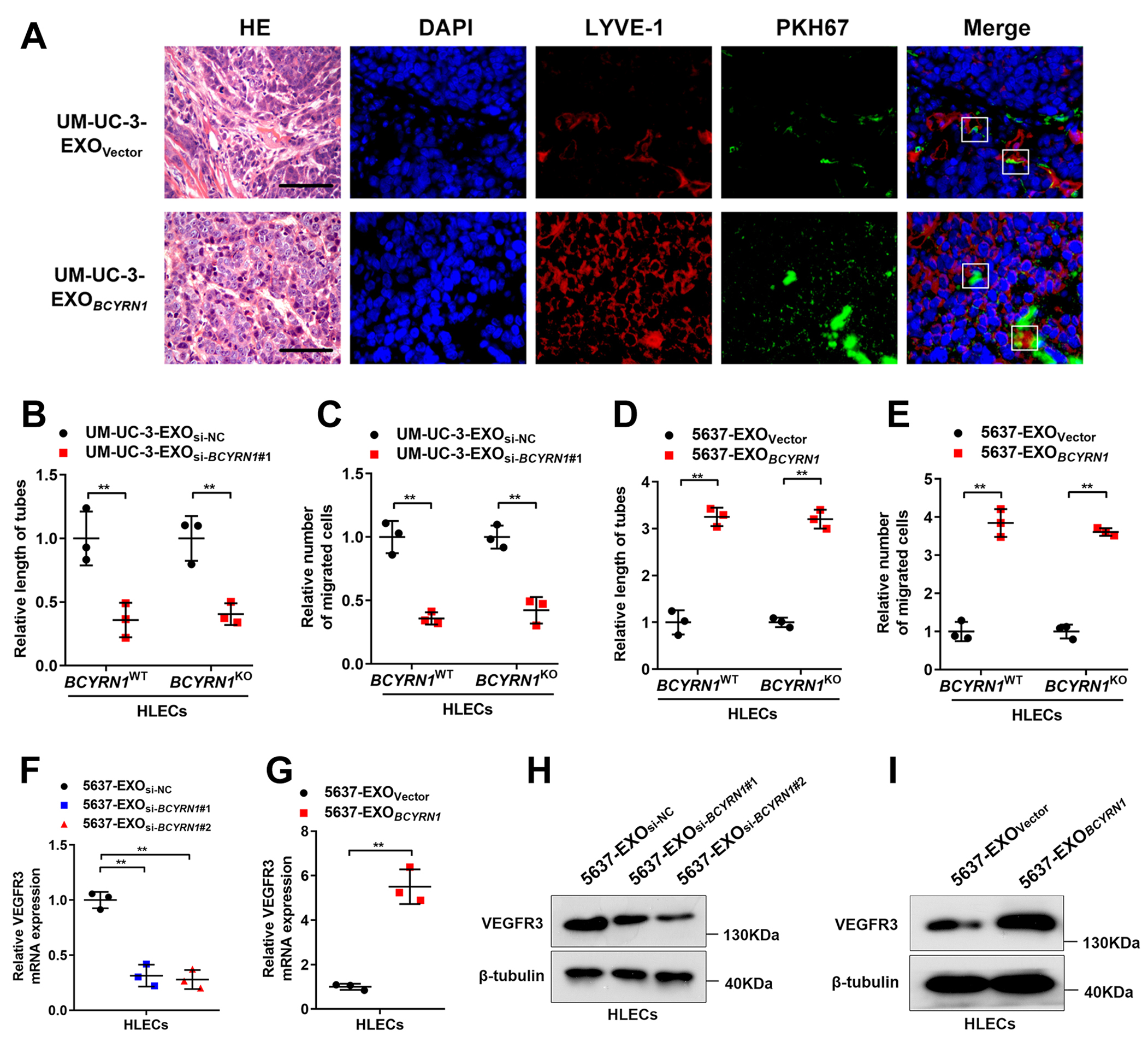


**FIGURE S8 Exosomal *BCYRN1* is internalized by lymphatic endothelial cells and upregulates VEGFR3 expression in HLECs. (A)** Representative fluorescence images of the internalization of PKH67-labeled UM-UC-3-EXO_Vector_ or UM-UC-3-EXO*_BCYRN1_* by LYVE-1-indicated lymphatic endothelial cells in the footpad primary tumor tissues. Scale bars: 50 μm. **(B-E)** Quantification of tube formation and Transwell migration of *BCYRN1*^WT^ or *BCYRN1*^KO^ HLECs treated *BCYRN1*-silenced UM-UC-3 or *BCYRN1*-overexpressing 5637 cell-secreted exosomes. **(F and G)** qRT-PCR analysis of VEGFR3 expression in HLECs treated with *BCYRN1*-silenced or overexpressing 5637 cell-secreted exosomes. **(H and I)** Western blotting analysis confirmed the upregulation of VEGFR3 in HLECs by exosomal *BCYRN1*. The statistical difference was assessed through Two-tailed Student’s *t* test in **B-E** and **G**; and one-way ANOVA followed by Dunnett’s tests in **F**. Error bars showed the standard deviations derived from three independent experiments. **p* < 0.05; ***p* < 0.01.


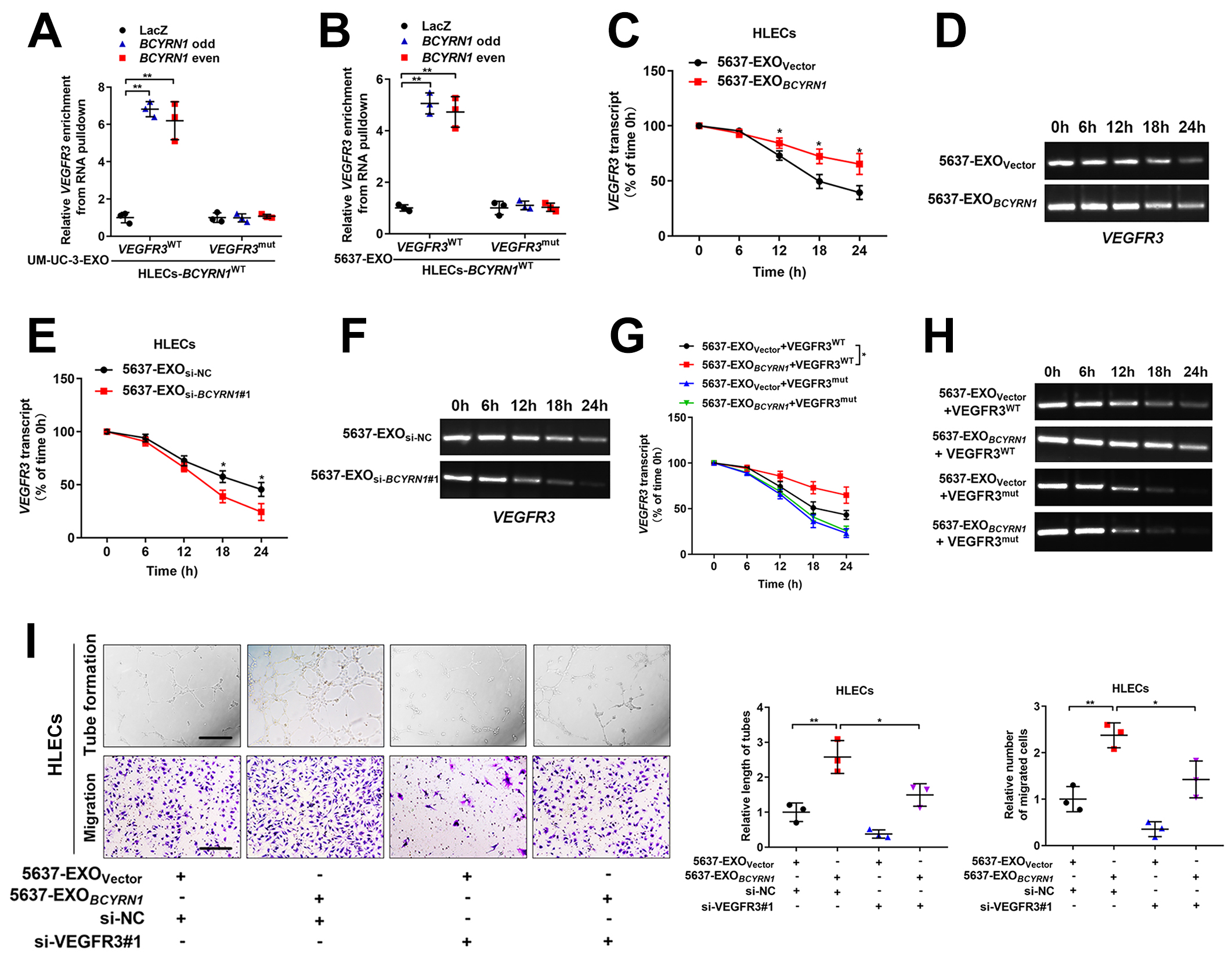


**FIGURE S9 Exosomal *BCYRN1* promotes VEGFR3 mRNA stability in HLECs to induce the lymphangiogenesis of BCa. (A and B)** RNA pull-down assays with biotin-labeled *BCYRN1* probe in BCa cell-secreted exosomes-treated *BCYRN1*^WT^ HLECs with or without mutating the VEGFR3 3’-UTR. **(C-F)** Actinomycin D assays for VEGFR3 mRNA in HLECs treated with *BCYRN1*-overexpressing or silenced 5637 cell-secreted exosomes. Quantification and representative images of agarose electrophoresis were showed. **(G and H)** Actinomycin D assays for VEGFR3 mRNA in 5637-EV_Vector_ or 5637-EV*_BCYRN1_*-treated HLECs with or without *BCYRN1*-binding sites mutation on VEGFR3 3’-UTR. Quantification and representative images of agarose electrophoresis were showed. **(I)** Representative images and quantification of tube formation and Transwell migration for 5637-EXO_Vector_ or 5637-EXO*_BCYRN1_*-treated HLECs transfected with si-NC or si-VEGFR3#1. Scale bars: 100 μm. The statistical difference was assessed through one-way ANOVA followed by Dunnett’s tests in **A**, **B** and **I;** and Two-tailed Student’s *t* test in **C, E** and **G**. Error bars showed the standard deviations derived from three independent experiments. **p* < 0.05; ***p* < 0.01.


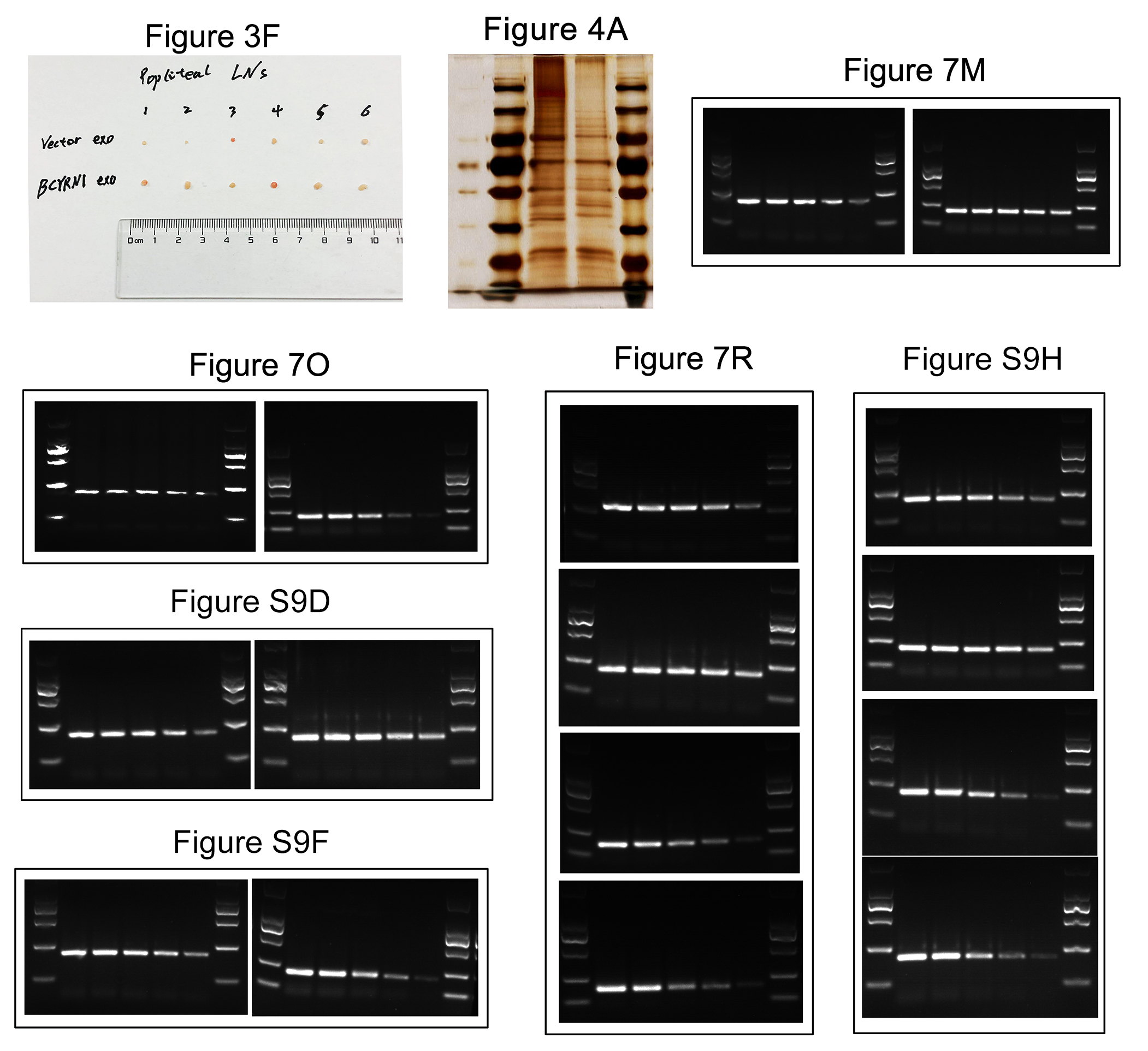


**FIGURE S10 Full uncut original pictures.**

**
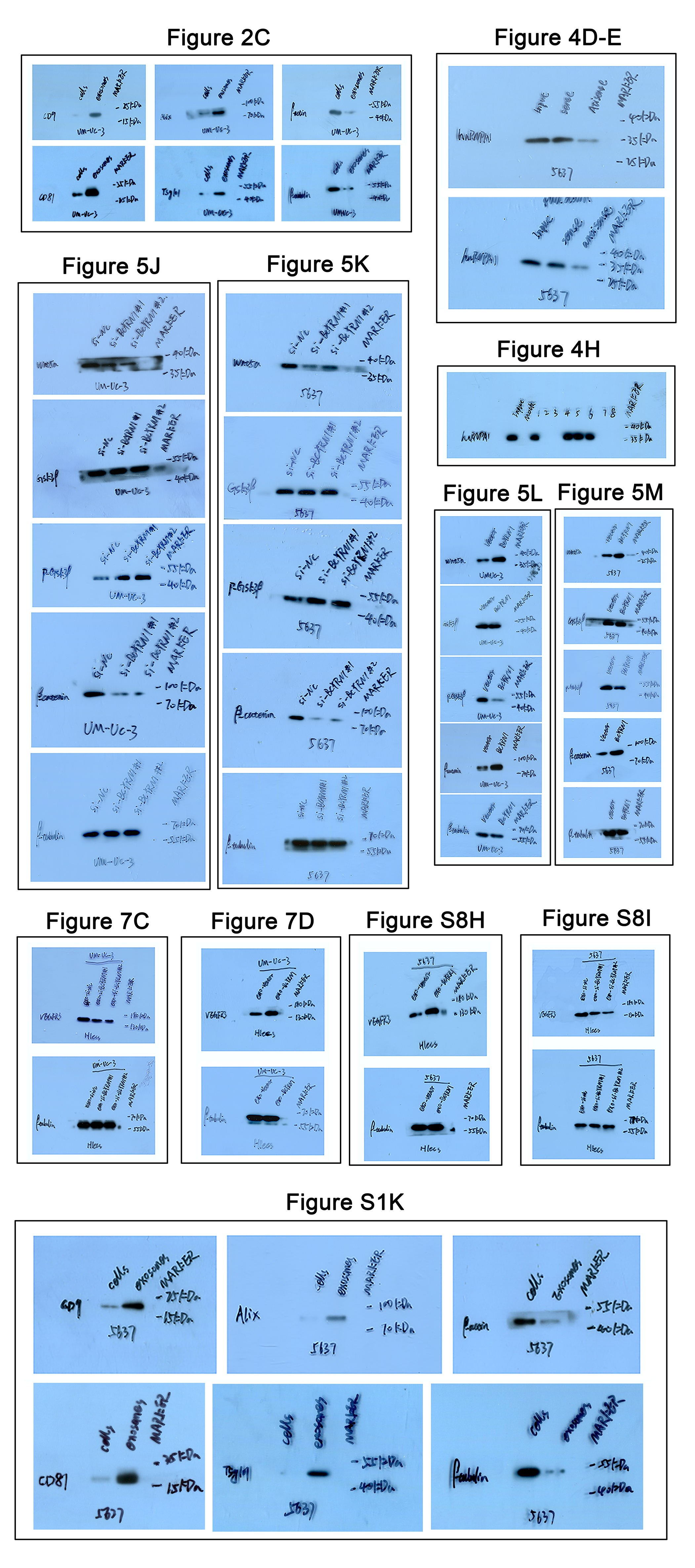
**

**FIGURE S11 Full uncut original pictures for western blotting analyzes.**

**Table S1. Correlation between *BCYRN1* and exosomal *BCYRN1* expressions and clinicopathologic characteristics in BCa patients**

| **Characteristics** | **No. of cases** | ***BCYRN1* expression** | | | **exosomal *BCYRN1* expression** | | |
| --- | --- | --- | --- | --- | --- | --- | --- |
|  |  | **Low** | **High** | ***p*-value**^†^ | **Low** | **High** | ***p*-value**^†^ |
| **Total cases** | 210 | 105 | 105 |  | 105 | 105 |  |
| **Gender** |  |  |  | 0.443 |  |  | 0.878 |
| Male | 151 | 78 | 73 |  | 76 | 75 |  |
| Female | 59 | 27 | 32 |  | 29 | 30 |  |
| **Age** |  |  |  | 0.572 |  |  | 0.396 |
| < 65 | 82 | 39 | 43 |  | 44 | 38 |  |
| ≥ 65 | 128 | 66 | 62 |  | 61 | 67 |  |
| **T stage** |  |  |  | **0.013^*^** |  |  | **0.029^*^** |
| T1 | 56 | 36 | 20 |  | 35 | 21 |  |
| T2-4 | 154 | 69 | 85 |  | 70 | 84 |  |
| **T grade** |  |  |  | 0.364 |  |  | 0.762 |
| Low | 62 | 28 | 34 |  | 30 | 32 |  |
| High | 148 | 77 | 71 |  | 75 | 73 |  |
| **Lymph node metastasis** |  |  |  | **0.001^**^** |  |  | **0.001^**^** |
| Negative | 157 | 103 | 54 |  | 102 | 55 |  |
| Positive | 53 | 2 | 51 |  | 3 | 50 |  |

Abbreviations: No. of Cases = number of cases; T stage = tumor stage; T grade = tumor grade.

^†^ Chi-square test, ^*^ *p* <0.05, ^**^ *p* <0.01.

**Table S2. Relative lncRNAs involving in the regulation of VEGFR3**

| **Gene Symbol** | **Location** | **Fold Change of VEGFR3** | |
| --- | --- | --- | --- |
| *MAP4K3-DT* | chr2: 39,514,146-39,599,539 | | 1.308901 |
| *RP5-857K21.7* | chr1: 632,757-633,438 | | 1.900057 |
| *BCYRN1* | chr2: 47,335,351-47,335,514 | | 2.985966 |

**Table S3. Effect of intratumoral injection of exosomal *BCYRN1* on popliteal LN metastasis *in vivo*.**

| **Xenograft** | **No. metastasis LNs** | **No. Non-metastasis LNs** | **Metastasis ratio** | ***P*-value**^†^ |
| --- | --- | --- | --- | --- |
| UM-UC-3-EXO_Vector_ | 4 | 8 | 33.33% | 0.013^*^ |
| UM-UC-3-EXO*_BCYRN1_* | 10 | 2 | 83.33% |

^†^ Chi-square test. ^*^ *p* <0.05, ^**^ *p* <0.01.

**Table S4. Popliteal LN metastasis ratio in different mice injected with exosomal *BCYRN1* on popliteal LN *in vivo*.**

| **Xenograft** | **No. metastasis LNs** | **No. Non-metastasis LNs** | **Metastasis ratio** | ***P*-value^a^** |
| --- | --- | --- | --- | --- |
| UM-UC-3-EXO_Vector_ | 1 | 11 | 8.33% | 0.025^*^ |
| UM-UC-3-EXO*_BCYRN1_* | 6 | 6 | 50% |

^a^ Chi-square test. ^*^ *p* <0.05, ^**^ *p* <0.01.

**Table S5. The possible TFO and TTS predicted by LongTarget for *BCYRN1* and *WNT5A* pro**moter.

| **Oligo ID** | **TFO (5’-3’)** | **Oligo ID** | **TTS (5’-3’)** |
| --- | --- | --- | --- |
| **TFO1** | CCTGTAATCCCAGCTCT | **TTS1** | GGATGCTAGTTTCTCCA |
| **TFO2** | CCGTTCTCCAGAAAAAG | **TTS2** | GCCAGTATTTCTAGACT |
| **TFO3** | GAAAAAAAAAAACAAAA | **TTS3** | CGTGTTTCATTCTCAGT |
| **TFO4** | ATCCCAGCTCTCAGGGA | **TTS4** | TTGGGACTAGACGGTTT |
| **TFO5** | GCGGTGGCTCACGCCTG | **TTS5** | CACCGTCCTCTGAAATA |

Abbreviations: TFO, Triplex-forming oligos; TTS, triplex target sites.

**Table S6. The popliteal LN metastasis of nude mice in different treated group *in vivo*.**

| **Xenograft** | **No. metastasis LNs** | **No. Non-metastasis LNs** | **Metastasis ratio** | ***P*-value**^†^ |
| --- | --- | --- | --- | --- |
| UM-UC-3-EXO_Vector_+PBS | 3 | 9 | 25% | 0.014^*^ |
| UM-UC-3-EXO*_BCYRN1_*+PBS | 9 | 3 | 75% |  |
| UM-UC-3-EXO_Vector_+SAR131675 | 1 | 11 | 8.33% | 0.041^*^ |
| UM-UC-3-EXO*_BCYRN1_*+SAR131675 | 4 | 8 | 33.33% |  |

^†^ Chi-square test. ^*^ *p* <0.05, ^**^ *p* <0.01.

**Table S7. Univariate and multivariate analysis of OS for exosomal *BCYRN1* expression in BCa patients (*n* = 210)**

| **Variables** | **Univariate analysis** | | | **Multivariate analysis** | | |
| --- | --- | --- | --- | --- | --- | --- |
|  | **HR** | **95%CI** | ***p*-value**^†^ | **HR** | **95%CI** | ***p*-value**^†^ |
| Age (<65 vs. ≥65) | 0.968 | 0.648-1.446 | 0.873 |  |  |  |
| Gender (Male vs. Female) | 1.199 | 0.768-1.873 | 0.425 |  |  |  |
| T stage (T2-4 vs. T1) | 1.119 | 0.716-1.748 | 0.621 |  |  |  |
| T grade (High vs. Low) | 0.873 | 0.580-1.341 | 0.514 |  |  |  |
| Lymph node metastasis (Positive vs. Negative) | 1.999 | 1.321-3.023 | **0.001^**^** | 1.626 | 1.002-2.637 | **0.049^*^** |
| Exosomal *BCYRN1* expression (High vs. Low) | 1.781 | 1.199-2.646 | **0.004^**^** | 1.436 | 0.906-2.278 | 0.124 |

Abbreviations: HR = hazard ratio; 95%CI = 95% confidence interval; T stage = tumor stage; T grade = tumor grade. ^†^ Cox regression analysis, ^*^ *p* <0.05, ^**^ *p* <0.01.

**Table S8. Univariate and multivariate analysis of DFS for exosomal *BCYRN1* expression in BCa patients (*n* = 210)**

| **Variables** | **Univariate analysis** | | | **Multivariate analysis** | | |
| --- | --- | --- | --- | --- | --- | --- |
|  | **HR** | **95%CI** | ***p*-value**^†^ | **HR** | **95%CI** | ***p*-value**^†^ |
| Age (<65 vs. ≥65) | 0.880 | 0.608-1.275 | 0.500 |  |  |  |
| Gender (Male vs. Female) | 1.210 | 0.803-1.823 | 0.361 |  |  |  |
| T stage (T2-4 vs. T1) | 1.152 | 0.737-1.802 | 0.535 |  |  |  |
| T grade (High vs. Low) | 0.845 | 0.581-1.227 | 0.375 |  |  |  |
| Lymph node metastasis  (Positive vs. Negative) | 1.728 | 1.174-2.543 | **0.006^**^** | 1.472 | 0.938-2.310 | 0.093 |
| Exosomal *BCYRN1* expression (High vs. Low) | 1.563 | 1.091-2.239 | **0.015^*^** | 1.320 | 0.868-2.007 | 0.194 |

Abbreviations: HR = hazard ratio; 95%CI =95% confidence interval; T stage =tumor stage; T grade = tumor grade. ^†^ Cox regression analysis, ^*^ *p* <0.05, ^**^ *p* <0.01.

**Table S9. Primers and probes used in the experiments.**

| **Gene** | **Sequence (5’-3’)** | **Application** |
| --- | --- | --- |
| BCYRN1 | F: ACGCCTGTAATCCCAGCTC  R: TGCTTTGAGGGAAGTTACGC | qRT-PCR |
| MAP4K3-DT | F: CCCAGGGTGTGTGAGATTCT  R: TTCCAAGATCCAGGTGCCAT | qRT-PCR |
| RP5-857K21.7 | F: ACCGTCTGAACTATCCTGCC  R: AGATTAGTCCGCCGTAGTCG | qRT-PCR |
| U6 | F: CTCGCTTCGGCAGCACA  R: AACGCTTCACGAATTTGCGT | qRT-PCR |
| 18S rRNA | F: ACACGGACAGGATTGACAGA  R: GGACATCTAAGGGCATCACA | qRT-PCR |
| WNT9A | F: CCTCTATGCCATCTCCTCGG  R: TTCCTTGACGAACTTGCTGC | qRT-PCR |
| WNT5A | F: TCCTCCGTGTTGTGATGTGA  R: GATACGCTGCAACACCTCTG | qRT-PCR |
| WNT3A | F: GGACAAAGCTACCAGGGAGT  R: ACCATCCCACCAAACTCGAT  R: AGAAGGTGACAAGAGGCTCC | qRT-PCR |
| LRP6 | F: ATCTCCGGCGAATTGAAAGC  R: TCTACCCTCTCGACCTGTCA  R: AGAAGGTGACAAGAGGCTCC | qRT-PCR |
| LRP5 | F: CGCCAAGACAGACAAGATCG  R: CTTGGCCACATTCACAGCTT | qRT-PCR |
| FZD5 | F: TAGCGGTTTTGTGTTCAGCC  R: TGCCATCTCACCAGCCTAAA  R: AGAAGGTGACAAGAGGCTCC | qRT-PCR |
| RNF43 | F: AGTGGGGAGACTAGCACCTA  R: ATGAATCGGAGCCTAGCCTC  R: AGAAGGTGACAAGAGGCTCC | qRT-PCR |
| ZNRF3 | F: TCTGAAGACCCGCTCAAGAG  R: GAGACCACGACGAAGAAAGC  R: AGAAGGTGACAAGAGGCTCC | qRT-PCR |
| ACTB | F: TGGCACCACACCTTCTACAA  R: CCAGAGGCGTACAGGGATAG | qRT-PCR |
| VEGFR3 | F: CAAGATGTTTGCCCAGCGTA  R: GATCACCAGCTTGCTCACAG | qRT-PCR |
| VEGFR3-3’UTR | F: AGGAGAAAGAGCCACATCCC  R: ATATCCTGGAGTAACGCGCA | qRT-PCR |
| GAPDH | F: CATGAGAAGTATGACAACAGCCT  R: AGTCCTTCCACGATACCAAAGT | ChIRP |
| U1 | F: TGAAGGCGCTTTTCTCATGG  R: CAGGGGAAAGCACGAACG | RIP |
| WNT5A-P1 | F: TACACGAACTGCCTTGGACT  R: CTCCTAGGCAGACACTCCAG | ChIRP |
| WNT5A-P2 | F: CACAGCTGGCACTCTTAACC  R: GCTACCCAACCACAAACCTT | ChIRP |
| WNT5A-P3 | F: GGCCTGACCCATGCTAAGA  R: TTGTCTAGCCTCTCAGCCAG | ChIRP |
| WNT5A-P4 | F: CTTGGGACTAGACGGTTTTGT  R: ACTGGACCTCACATGACAAG | ChIRP |
| WNT5A-P5 | F: TTCATCCATTCGCCCATTCG  R: TGTGCTGAGATTATGCCCCA | ChIRP |
| *BCYRN1*-sgRNA1 | TGATGAGCTATATAACCCTA | CRISPR/Cas9 |
| *BCYRN1*-sgRNA2 | GCTGCTACTGCTATATGTGG | CRISPR/Cas9 |
| si-*BCYRN1*#1 | sense: GCUAAGAGGCGGGAGGAUATT  antisense: UAUCCUCCCGCCUCUUAGCTT | si-RNA |
| si-*BCYRN1*#2 | sense: GCGGUGGCUCACGCCUGUATT  antisense: UACAGGCGUGAGCCACCGCTT | si-RNA |
| si-MAP4K3-DT#1 | sense: GAGUUAUGACAUAACAGAAUU  antisense: UUCUGUUAUGUCAUAACUCUG | si-RNA |
| si-MAP4K3-DT#2 | sense: GAAGUUGUAGGGAUAAGAAAU  antisense: UUCUUAUCCCUACAACUUCUU | si-RNA |
| si-RP5-857K21.7#1 | sense: GACUAAUCUUCAACUCCUACA  antisense: UAGGAGUUGAAGAUUAGUCCG | si-RNA |
| si-RP5-857K21.7#2 | sense: CGUUGACAAUCGAGUAGUACU  antisense: UACUACUCGAUUGUCAACGUC | si-RNA |
| si-VEGFR3#1 | sense: GCAGCUACGUCUGCUACUATT  antisense: UAGUAGCAGACGUAGCUGCTT | si-RNA |
| BCYRN1_odds1 | GTGAGCCACCGCGCCCGGCC 3’-Biotin labeled | ChIRP |
| BCYRN1_even1 | GGAGAACGGGGTCTCGCTAT 3’-Biotin labeled | ChIRP |
| BCYRN1 | TTGAGGGAAGTTACGCTTATT  5’-DIG labeled and 3’-DIG labeled | ISH |
| U6 | CACGAATTTGCGTGTCATCCTT  5’-DIG labeled and 3’-DIG labeled | ISH |
| Scramble | GTGTAACACGTCTATACGCCCA  5’-DIG labeled and 3’-DIG labeled | ISH |

**Table S10. Antibodies used in the experiments.**

| **Product** | **Source** | **No. of Catalogue** |
| --- | --- | --- |
| **Primary antibody:** |  |  |
| ***Western blot:*** |  |  |
| anti-β-actin | Sigma-Aldrich | A5441 |
| anti-CD9 | Cell Signaling Technology | 13403 |
| anti-ALIX | Cell Signaling Technology | 92880 |
| anti-hnRNPA1 | Abcam | ab5832 |
| anti-WNT5A | Proteintech | 55184-1-AP |
| anti-GSK3β | Cell Signaling Technology | 5676S |
| anti-p-GSK3β | Cell Signaling Technology | 5558T |
| anti-β-catenin | Cell Signaling Technology | 8480S |
| anti-β-tubulin | CWBio | CW0098 |
| anti-VEGFR3 | absin | abs100388 |
| anti-TSG101 | Abcam | ab83 |
| anti-CD81 | Abcam | ab79559 |
| ***IHC:*** |  |  |
| anti-LYVE-1 | Abcam | ab218535 |
| anti-GFP | Abcam | ab183734 |
| anti-CD34 | Abcam | ab81289 |
| anti-Ki67 | Sino Biological | ZM-0166 |
| anti-VEGFR3 | absin | abs100388 |
| anti-WNT5A | Proteintech | 55184-1-AP |
| ***IF:*** |  |  |
| anti-hnRNPA1 | Abcam | ab5832 |
| anti-LYVE-1 | Abcam | ab218535 |
| ***IP:*** |  |  |
| anti-hnRNPA1 | Abcam | ab5832 |
| anti-H3K4me3 | Abcam | ab1012 |
| **Secondary antibody:** |  |  |
| ***Western blot:*** |  |  |
| anti-rabbit IgG-HRP | Cell Signaling Technology | 7074 |
| anti-mouse IgG-HRP | Cell Signaling Technology | 7076 |
| ***IHC:*** |  |  |
| anti-rabbit IgG-HRP | Proteintech | SA00001-2 |
| anti-mouse IgG-HRP | Proteintech | SA00001-1 |
| ***IF:*** |  |  |
| anti-rabbit IgG-HRP | Panovue Biological | 0015001010 |

# SUPPLEMENTARY METHODS

### IHC analysis

IHC analysis was performed to detect the target proteins in BCa tissues, nude footpad tumors, or popliteal LNs. Briefly, the sections were heated in an incubator at 60 °C for 2 h, deparaffinized using xylene, and rehydrated with gradient alcohol (95%, 85% and 70%, respectively). Then, the sections were submerged in EDTA buffer to retrieval the antigen in a microwave at medium high heat for 7 min and medium-low heat for 14 min, respectively. Next, to block the activity of endogenous peroxidase, the samples were incubated with 3% hydrogen peroxide for 15 min, followed by goat serum for 30 min at room temperature. Subsequently, the sections were treated with primary antibody at 4 ℃ overnight and corresponding secondary antibodies at room temperature for 30 min, respectively. After staining with 3,3'-Diaminobenzidine (DAB) and counterstaining with hematoxylin, the sections were put on a Nikon eclipse 80i microscope (Nikon, Tokyo, Japan) to capture images.

### ISH anlaysis

ISH analysis was conducted to detect the expression of *BCYRN1* in BCa tissue specimens through the ISH Detection kitⅢ (Boster Biological Technology Co., Ltd, California, USA) with the instructions of manufactures. Briefly, the sections were treated with pepsin to thoroughly digest the tissues after deparaffinized and rehydrated as the same steps described in IHC analysis. Then, the sections were hybridized with the digoxin labeled-*BCYRN1*oligonucleotide probe at 37℃ overnight, followed with the incubation of anti-digoxin antibody at 37℃ for 2h. The BCIP/NBT was used to stain the sections and nuclear fast red was used for the counterstaining. And the images were recorded by Nikon eclipse 80i (Nikon, Tokyo, Japan). The probes used in this assay were listed in Table S9.

### Cytosolic and nuclear fraction

To detect the cell location of *BCYRN1*, the cytosolic and nuclear fraction experiments were conducted with PARISTM Kit (Invitrogen) under the instruction of manufacturers. 18sRNA and U6 were used as internal references.

### Immunofluorescence (IF)

The IF analysis was performed as follow: briefly, the cells were seeded in 5cm confocal dish and incubated for 24h. Then, removing the supernatant and washing the cells with PBS for 3 times, after which 4% paraformaldehyde was used to fix the cells for 15 min. Next, the treatment with 0.1% Triton X-100 for 20 min was carried out to permeabilize the cells followed by the blocking with goat serum for 30 min. Subsequently, the primary antibody was supplemented to incubate with the cells at 4℃ overnight followed with the treatment of the corresponding secondary antibody for 1 h. Finally, the DAPI was used to stain the nuclei for 10 min and the images were recorded by a LSM710 confocal microscopy (Zeiss, Pleasanton, CA, USA).

### FISH

As for the FISH analysis, the cells seeded in in the confocal dish were pretreated using 0.5% Triton X-100 to permeabilize the cells as describing in the IF assays. Then, the *BCYRN1* probes labeled with Alexa Fluor 555 (GenePharma, Shanghai, China) were used to hybridize the cells at 4 °C overnight followed by the staining of cell nuclei with DAPI at room temperature for 10 min. The images were captured with LSM710 confocal microscopy (Zeiss, Pleasanton, CA, USA).

### Isolation of exosomes

To isolate the exosomes from the culture media of BCa cells, gradient centrifugation was conducted. Briefly, the culture media supplemented with 10% exosome-free FBS was used to culture the BCa cells for 72 h, after which the supernatant from indicated cells was collected and centrifuged at 2000 × *g* for 15 min to discard the cell debris. Subsequently, the supernatant was put into a new tube for further centrifugation at 10,000 × *g* for 30 min, followed by ultracentrifugation at 120,000 × *g* for 75 min in an Optima XE-100 ultracentrifuge (Beckman Coulter, Brea, CA, USA). The exosomal precipitate was thoroughly resuspended in moderate PBS to obtain the exosomes suspension, which was preserved in a -80 °C freezer for further analysis. The method of isolating the exosomes from urine specimens was the same as that described above. All centrifugations were performed at 4 °C.

### Transmission electron microscopy

To identify the characteristics of isolated exosomes, we performed the transmission electron microscopy in which the exosomes were resuspended and fixed by 4% polyformaldehyde. Then, the exosomal suspension was placed on copper grids for 60 min followed with the fixation with 2.5% glutaraldehyde for 10 min. Subsequently, the PBS was used to wash the grids for 5 times and uranyl acetate was used to counterstain the exosomes for 5 min. After air-drying at room temperature, the grids were placed on a Hitachi transmission electron microscope (Hitachi, Tokyo, Japan) for capturing the images.

### Lentivirus infection and cell transfection

For lentivirus infection, we first packaged the full-length of *BCYRN1* into a lentivirus vector (GenePharma, Shanghai, China), which was then transfected into HEK-293T cells to amplify the target virus. Then, the lentiviruses were harvested and purified to infect UM-UC-3 and 5637 cells to construct the *BCYRN1* stable overexpression cell lines, in which puromycin (Sigma Aldrich, St. Louis, MO, USA) was used to select the successfully infected cells for 2 weeks.

During cell transfection, short interfering RNA (siRNA) oligonucleotides for the target gene and the pcDNA3.1 expression vector with the target gene were purchased from GenePharma company (Shanghai, China) and were used to knock down or overexpress the target gene in UM-UC-3 and 5637 cells, respectively. All the transfections were conducted with assistance of lipofectamine 3000 (Invitrogen, Carlsbad, CA, USA) under the protocols from manufacturers. qRT-PCR analysis was used to examine the efficiency of transfection.

### Cell proliferation assays

To determine the regulatory role of *BCYRN1* in BCa cells proliferation, the Cell Counting Kit 8 (CCK-8) assays and 5-ethynyl-20-deoxyuridine (EdU) assays were performed with si-*BCYRN1* transfected BCa cells.

As for CCK-8 assays, 1000 cells per well supplemented in 100 μl culture medium were seeded into 96-well plates, after which the CCK-8 kit (APExBIO, USA) was used to evaluate the cell growth by determining the absorbance at OD450 nm with a microplate reader (Epoch, BioTek, USA) at 0, 24, 48, 72, and 96 h under the instruction of manufacturers.

As for EdU assays, BCa cells after transfection were seeded into 12 well plates and cultured for 24 h, after which the cells were stained with EdU and Hoechst following the instructions of EdU kit (RioboBio, Guangzhou, China) and the images were captured with an Olympus laser scanning microscope system (Tokyo, Japan).

### Tube formation assays

Briefly, a total of 300 µl of a mixture of growth factor reduced Matrigel (BD Biosciences, San Jose, CA, USA) and FBS-free ECM (1:2, v/v) were placed in the wells of a 24-well plate and incubated at 37 ℃ overnight. Then, the HLECs incubated with equivalent amounts of PBS, BCa cell culture media or 10 μg/ml BCa secreted-exosomes for 48 h were digested and resuspended in fresh culture media, and seeded into the 24 well plates for 1 × 10^5^ cells per well. The images of the formed lymphatic vessels were captured using inverted fluorescence microscopy and Image J software (NIH, Bethesda, MD, USA) was used to analyze the length of the tubes.

### Transwell assays

Transwell assays were carried out to evaluate the migration ability of HLECs treated with BCa culture media or isolated exosomes. Briefly, after the incubation with PBS, BCa culture media or 10 μg/ml isolated exosomes for 48 h, the HLECs were harvested and resuspended in FBS-free media. Subsequently, 300 μl suspension contained 1 × 10^5^ cells was seeded into the upper chamber (24-well insert, 8 μm, Corning Costar Corp, NY, USA) and 700 μl culture media supplemented with 5% FBS was added to the lower chamber. The whole system was incubated in a humidified incubator with 5% CO_2_ at 37°C for 14 h, after which the cells were fixed in 4% paraformaldehyde for 15 min and stained with crystal violet for another 15 min. The inverted microscope was used to capture the images and the Image J software was used to count the migrated cells at five random fields.

### Subcutaneous tumorigenicity model

The mice (about 4–5 weeks old BALB/c nude mice) used to construct the subcutaneous tumorigenicity model were purchased form the Experimental Animal Center, Sun Yat-sen University (Guangzhou, China). Briefly, 5 × 10^6^ UM-UC-3 cells transfected with sh-NC or sh-*BCYRN1*#1 were injected into the right hind legs of mice, after which the primary tumors were resected and embedded with paraffin for further analysis. The animal experiments were conducted under the approvement of the Institutional Animal Care and Use Committee of Sun Yat-sen University.

### RNA extraction and qRT-PCR analysis

The extractions of total RNA from tissues, BCa cells and the corresponding exosomes were conducted through the TRIZOL Reagent (Life Technologies) under the instructions of manufacturers. Subsequently, the extracted total RNA was reversely transcribed into cDNA with Prime Script TM RT Master Mix (Takara, Shiga, Japan), which was further analyzed by qRT-PCR assays to determinate the expression of target gene on a Bio-Rad CFX96 system with TBGreen II (Takara). And the 2^-∆CT^ method was used to evaluate the relative expression level, in which GAPDH or ACTB were used as internal control. The details of primers used in this study were shown in Table S9.

### Western blotting analysis

Western blotting analysis was performed to determinate the target proteins expression in BCa cells and HLECs. Briefly, the pretreated cells were thoroughly lysed in the RIPA lysis buffer contained with 1% phosphatase inhibitor and 1% protease inhibitor, followed with the centrifugation at 4°C for 30 min to obtain the total proteins. Then, the concentration of proteins was measured by BCA Protein Assay Kit (Thermo Fisher Scientific, Carlsbad, CA, USA). Next, 10% SDS-PAGE gel was used to separate the total proteins which were then transferred into a polyvinylidene fluoride membrane (Millipore, Billerica, MA). After blocking with 5% BSA at room temperature for 1 h, the membrane was incubated with primary antibodies at 4°C overnight, followed with the incubation with corresponding secondary antibodies at room temperature for 1 h. The target proteins were detected by ELC reagent (Thermo Fisher Scientific, Rochester, NY).

### RNA pull-down assays

For the pulldown of RNAs, the biotinylated *BCYRN1* and antisense sequences were obtained as described in pulldown of proteins, which were incubated with cell lysate at 4 °C overnight. Then, the prewashed beads were added to capture the *BCYRN1*-binding RNAs, followed by further analysis using qRT-PCR.

### RIP assays

EZ-Magna RIP kit (Millipore) was used to conduct the RIP assays according to the manufacturer’s protocols. Briefly, 1× 10^7^ UM-UC-3 cells were lysed completely by RIP lysis buffer and then were immunoprecipitated with magnetic beads conjugated with anti-hnRNPA1 antibody (Abcam, MA, USA) or rabbit anti-IgG as a negative control. The precipitated RNAs were eluted and further analyzed by qRT-PCR assays in which U1 was used as a non-specific control.

### Serial deletion analysis

To determine the essential sequence of *BCYRN1* involved in its interaction with hnRNPA1, the Serial deletion analysis was performed, in which pGSI vectors contained with different truncate *BCYRN1* fragment were constructed and further used to conduct RNA pulldown assays.

### Dual-luciferase reporter assays

To determinate the mechanism of *BCYRN1* in the transcriptional activation of *WNT5A*, the Dual-luciferase reporter assays were carried out with the Dual-Luciferase Reporter Assay System (Promega, Madison, WI, USA) under the instructions of manufacturers. Briefly, the different pretreated cells were harvested and washed with PBS for 3 times, which were further lysed in PBL lysis buffer for 20 min. Then the supernatants were collected and added into 96-well plates for10 μl per well, followed with 100 μl Luciferase Assay Reagent II for exactly 2 seconds. Subsequently, the luciferase intensity was recorded by a MK3 plates reader (Thermo, Shanghai, China) and Renilla luciferase intensity was detected as controls.

### TOP-flash/FOP-flash luciferase reporter assays

To evaluate the β-catenin signaling activity after silencing or overexpressing *BCYRN1* in BCa cells, the TOP-flash/FOP-flash luciferase reporter assays were conducted. Briefly, BCa cells were cultured in six-well plates and transfected with siRNA or pcDNA3.1 expression vector targeting *BCYRN1* to downregulate or upregulate *BCYRN1* expression, respectively. And then the transfection of TOP-flash or FOP-flash plasmids (GenePharma, Shanghai, China) was performed, among which the FOP-flash was used as a negative control. The luciferase activity was detected with the Dual-Luciferase Reporter Assay System mentioned above and Renilla reporter luciferase was used to normalize the efficiency of transfection.

### ELISA

For the quantitation of secreted VEGF-C, ELISA assays were performed by Human VEGF-C ELISA Kit (Abcam) under the instructions of manufacturers. The culture media from *BCYRN1*-overexpressing or silencing BCa cells were collected and added into the appropriate wells followed with the incubation of VEGF-C antibody at room temperature for 1 h. Subsequently, the plates were washed with PBST for 5 times and treated with TMB Development Solution for 10 min, after which 100 µL Stop Solution per wells was added to stop the reaction and the OD at 450 nm were measured with a SYNERGY H1 microplate handler (Bio-Tek, USA).

### Internalization analysis of exosomes

The exosomal internalization assays were carried out to confirm the internalization of BCa cell secreted exosomes by HLECs. Briefly, we labelled the isolated exosomes with PKH67 dye using a PKH67 green fluorescent labelling kit (Sigma-Aldrich) for 5 min and then 5% bovine serum albumin (BSA) was added to neutralize the remaining dye, after which ultracentrifugation was conducted to reprecipitate the exosomes labelled with PKH-67. The reprecipitated exosomes were resuspended in PBS and 10 μg/ml exosomes were incubated with HLECs for 12 h in an incubator at 37 °C with 5% CO2. Subsequently, the PKH-67-labelled exosome-treated HLECs were fixed with 4% polyformaldehyde for 15 min and then 4′,6-diamidino-2-phenylindole (DAPI) was used to stain the nuclei for 5 min. The internalization of BCa cell-secreted exosomes by HLECs was observed and the images were captured under a Zeiss confocal microscope system (Zeiss, Wetzlar, Germany).

As for the analysis of the internalization of BCa cell-secreted exosomal *BCYRN1* by lymphatic endothelial cells *in vivo*, the UM-UC-3-EXO_Vector_ or UM-UC-3-EXO*_BCYRN1_* were labelled with PKH67 as mentioned above and 10 μg indicated exosomes in 20 μL PBS were injected intratumorally into the footpad tumors of mice. And then the footpad tumors of mice were resected for detecting the uptake of PKH67-labelled exosomes by lymphatic endothelial cells. The lymphatic endothelial cells were indicated by the staining of anti-LYVE-1 antibody and the co-location of PKH67-labelled exosomes and lymphatic endothelial cells was recorded by Zeiss confocal microscope system (Zeiss, Wetzlar, Germany).

### Actinomycin assays

The Actinomycin assays were conducted to detect the mechanism of exosomal *BCYRN1* in the regulation of VEGFR3 expression. Briefly, the HLECs were seeded into the 12 well plate followed by the incubation with 10 μg/ml exosomes secreted by indicated BCa cells. Then, the actinomycin D was supplemented and the cells were harvested for RNAs extraction at 0, 6, 12, 18, 24 h, respectively. And the expression of VEGFR3 in different time was assessed by qRT-PCR analysis.

### Bioinformatic analysis

The clinical association of *BCYRN1* in various cancers was revealed through analysis of The Cancer Genome Atlas (TCGA) database at GEPIA (<http://gepia.cancer-pku.cn/index.html>). The [secondary](https://cn.bing.com/dict/search?q=secondary&FORM=BDVSP6&mkt=zh-cn) [structure](https://cn.bing.com/dict/search?q=structure&FORM=BDVSP6&mkt=zh-cn) of *BCYRN1* and the binding motif of hnRNPA1 were acquired from RNAalifold (<http://rna.tbi.univie.ac.at/cgi-bin/RNAWebSuite/RNAalifold.cgi>) and POSTAR2 (<http://lulab.life.tsinghua.edu.cn/postar2>), respectively.
